# Protective Effect of Allergen Immunotherapy in Patients With Allergic Rhinitis and Asthma Against COVID-19 Infection: Observational, Nationwide, and Multicenter Study

**DOI:** 10.2196/50846

**Published:** 2024-10-16

**Authors:** Rundong Qin, Yan Feng, Huanping Zhang, Beibei Zhao, Wei Lei, Hongying Sun, Lili Zhi, Zhongsheng Zheng, Siqin Wang, Yafeng Yu, Shengxue Jiang, Changshan Liu, Xingkai Ma, Hui Ma, Huiying Wang, Hang Lin, Qiaojie He, Lingying Wu, Yingying Zhai, Honglue Lu, Shi Chen, Yan Ma, Xiaohong Jin, Shan Deng, Nanshan Zhong, Ruchong Chen, Jing Li

**Affiliations:** 1 Department of Allergy and Clinical Immunology, State Key Laboratory of Respiratory Disease, National Clinical Research Center for Respiratory Disease, Guangzhou Institute of Respiratory Health, The First Affiliated Hospital of Guangzhou Medical University, Guangzhou, 510120, China, 86 189 2886 8259, 86 20 3429 8996; 2 Department of Otolaryngology Head and Neck Surgery, The First Hospital, Shanxi Medical University, Taiyuan, China; 3 Department of Allergology, Shanxi Bethune Hospital, Shanxi Academy of Medical Science, Tongji Shanxi Hospital, Third Hospital of Shanxi Medical University, Shanxi, China; 4 Department of Allergy, The Second Affiliated Hospital of Dalian Medical University, Liaoning, China; 5 Department of Pulmonary and Critical Care Medicine, The First Affiliated Hospital of Soochow University, Suzhou, China; 6 Department of Pulmonary and Critical Care Medicine, The First Hospital of Jilin University, Changchun, China; 7 Department of Allergy, Shandong Provincial Qianfoshan Hospital, Shandong Institute of Respiratory Diseases, The First Affiliated Hospital of Shandong First Medical University, Jinan, China; 8 Department of Respiratory and Critical Care Medicine, The First Affiliated Hospital of Shantou University Medical College, Shantou, China; 9 Department of Allergy, Henan Provincial People’s Hospital, Zhengzhou University People’s Hospital, Zhengzhou, China; 10 Department of Otolaryngology, The First Affiliated Hospital of Soochow University, Suzhou, China; 11 Department of Allergy, Shengjing Hospital of China Medical University, Liaoning, China; 12 Department of Pediatrics, The Second Hospital of Tianjin Medical University, Children’s Respiratory and Asthma Research Center of Tianjin Medical University, Tianjin, China; 13 Department of Otorhinolaryngology, The Affiliated Zhangjiagang Hospital of Soochow University, Zhangjiagang, China; 14 Department of Respiratory and Critical Care Medicine, Chest Hospital, Tianjin University, Tianjin, China; 15 Department of Allergy and Clinical Immunology, The Second Affiliated Hospital of Zhejiang University School of Medicine, Hangzhou, China; 16 Department of Allergy, The Affiliated Hospital of Qingdao University, Qingdao, China; 17 Department of Allergy, The Second Affiliated Hospital of Dalian Medical University, Dalian, China; 18 Department of Allergy, The Third People’s Hospital of Datong, Datong, China; 19 Department of Pediatrics, The First Affiliated Hospital of Guangzhou Medical University, Guangzhou, China; 20 Department of Otolaryngology, Suzhou Hospital, Affiliated Hospital of Medical School, Nanjing University, Suzhou, China; 21 Center for Prevention and Treatment of Pediatric Asthma, Hainan General Hospital, Hainan Affiliated Hospital of Hainan Medical University, Haikou, China; 22 Department of Otorhinolaryngology Head and Neck Surgery, The First Affiliated Hospital of Anhui Medical University, Hefei, China; 23 Department of Pediatrics, Taizhou Hospital of Zhejiang Province Affiliated to Wenzhou Medical University, Taizhou, China; 24 Department of Pulmonary and Critical Care Medicine, State Key Laboratory of Respiratory Disease, National Clinical Research Center for Respiratory Disease, Guangzhou Institute of Respiratory Health, The First Affiliated Hospital of Guangzhou Medical University, Guangzhou, China

**Keywords:** allergen immunotherapy, COVID-19, antiviral effect, allergic rhinitis, asthma, viral infection, allergic disease, trajectory, questionnaire-based survey, clinical evidence

## Abstract

**Background:**

Allergic diseases are associated with an increased susceptibility to respiratory tract infections. Although allergen immunotherapy (AIT) alters the course of allergies, there is limited evidence from clinical practice demonstrating its ability to enhance the host defense against pathogens.

**Objective:**

The aim of this study was to investigate the protective effect of AIT against viral infection in patients with allergic rhinitis (AR) and allergic asthma (AS) based on clinical evidence.

**Methods:**

A multicenter, questionnaire-based survey was conducted during a tremendous surge in COVID-19 cases between February 10, 2023, and March 15, 2023, in 81 centers across China recruiting healthy volunteers and patients with AR and AS to investigate the clinical outcomes of COVID-19 infection.

**Results:**

Of 10,151 participants recruited in the survey, 3654 patients and 2192 healthy volunteers who tested positive for COVID-19 were included in this analysis after screening. Overall, no significant differences in COVID-19 outcomes were observed between patients and healthy volunteers. An additional 451 patients were excluded due to their use of biologics as the sole add-on treatment, leaving 3203 patients in the further analysis. Of them, 1752 were undergoing routine medication treatment (RMT; the RMT group), whereas 1057 and 394 were receiving AIT and a combination of AIT and omalizumab (OMA) as adjunct therapies to RMT, respectively (AIT+RMT and AIT+OMA+RMT groups). The AIT group showed milder COVID-19 symptoms, shorter recovery periods, and a lower likelihood of hospitalization or emergency department visits than the RMT group (all *P*<.05). After adjusting for confounding factors, including demographic characteristics and COVID-19 vaccination, AIT remained a significant protective factor associated with shorter recovery time (adjusted odds ratio [OR] 0.62, 95% CI 0.52‐0.75; adjusted *P*<.001) and a lower incidence of hospitalization or emergency department visits (adjusted OR 0.73, 95% CI 0.54‐0.98; adjusted *P*=.03). Furthermore, the AIT+OMA+RMT group showed greater protection with a shorter recovery time (adjusted OR 0.51, 95% CI 0.34‐0.74; adjusted *P*<.001) than the AIT+RMT group.

**Conclusions:**

Our multicenter observational study provides valuable clinical evidence supporting the protective effect of AIT against COVID-19 infection in patients with AR and AS.

## Introduction

Allergic rhinitis (AR) and allergic asthma (AS) are prevalent chronic respiratory conditions characterized by dysregulated immune responses in the airways, which play a significant role in their pathogenesis [1,2]. Therefore, the restoration and modulation of immune functions have emerged as essential strategies, such as allergen immunotherapy (AIT) [1,3].

AIT is a therapeutic approach used for allergic diseases that can modulate the immune response and potentially alter the course of allergic conditions [4,5]. Robust evidence from randomized controlled trials (RCTs) and meta-analyses supports the effectiveness of AIT in reducing allergy symptoms, decreasing daily medication dosage, and mitigating exacerbation [6,7]. The underlying mechanism of AIT involves restoring immune homeostasis by shifting the predominance from a Th2 response, which is responsible for allergic inflammation, toward a more harmonized Th1/Th2 response [8,9]. The Th1 response is associated with innate immunity against various pathogens, including viruses and bacteria [10]. A recent double-blind RCT demonstrated that AIT significantly enhanced the bronchial epithelial antiviral resistance to viral infection in patients with AS after 24 weeks of treatment [11]. Nevertheless, the available clinical data are still insufficient to firmly support the hypothesis that immune modifications induced by AIT can effectively improve the host defense against pathogens.

COVID-19, caused by SARS-CoV-2, has emerged as a global pandemic with a significant mortality rate [12,13]. The disease particularly affects individuals with pre-existing respiratory conditions [14,15]. Interestingly, AR and AS have been observed to confer a protective effect against COVID-19 infection [16]. Although the precise underlying mechanisms remain incompletely understood, it is hypothesized that attachment and entry of SARS-CoV-2 are hindered by the downregulation of angiotensin-converting enzyme 2 in the airways of individuals who are allergic [17]. Moreover, emerging evidence suggests that biologics, such as omalizumab (OMA), an anti-immunoglobulin E medication, may provide protection against COVID-19 infection in patients with allergies by restoring interferon (IFN) production [18]. Nevertheless, there are currently limited available data regarding the association between AIT and its potential protective effects against COVID-19 in individuals with AR or AS [19].

Over a 2-month period starting from December 7, 2022, China experienced a significant upsurge in COVID-19 cases, creating a unique window to investigate the potential advantages of AIT in strengthening innate immunity against viral infections in a clinical setting, as opposed to relying solely on RCTs. We conducted this study to explore the protective efficacy of AIT in enhancing clinical outcomes for individuals with AR and AS who contracted COVID-19 infection. This evaluation involved a multicenter survey using a questionnaire-based approach. Additionally, we explored whether the combined therapy of AIT and OMA (AIT+OMA) exerted an augmented antiviral effect.

## Methods

### Study Design

We conducted a questionnaire-based survey between February 10, 2023, and March 15, 2023, across 81 allergy centers in China to examine the differential impact of COVID-19 infection on individuals with AR and AS. This study included patients who met the following inclusion criteria: (1) patients aged between 6 and 60 years, (2) patients with a confirmed diagnosis of AR and AS, (3) patients undergoing routine medication treatment (RMT), and (4) patients receiving AIT for at least 1 year or receiving OMA for at least 4 months as adjunct treatments to RMT. RMT comprised the use of nasal or inhaled corticosteroids (ICSs) and oral drugs, following the relevant guidelines [1,3]. Regarding the different types of corticosteroids, the dosages were converted using budesonide equivalence. Furthermore, healthy volunteers without any acute or chronic illnesses were recruited through community-based advertisements. All eligible participants, including patients and healthy volunteers, were invited to participate in the survey and complete a unified questionnaire that collected demographic, clinical, and COVID-19–related information. In cases where child participants could not independently complete the questionnaire, adult family members were allowed to fill it out on their behalf. Participants were fully informed about the study objectives and procedures and willingly agreed to participate.

### Ethical Considerations

This study was approved by the ethics review board (IRB: 2022‐76) of the leading center, the First Affiliated Hospital of Guangzhou Medical University. Informed consent was obtained from all participants prior to their inclusion in the study. Privacy and confidentiality of participants were maintained throughout the study, and all data were anonymized to protect individual identities. Participants were provided with information about the study’s purpose, procedures, and potential risks, and they voluntarily agreed to participate. No compensation was provided for participation in this study.

### Questionnaire

The questionnaire consisted of 3 main sections, viz, demographic, clinical, and COVID-19–related information sections. The first section collected demographic characteristics, such as age, sex, height, and weight. The second section focused on clinical details, including smoking history, disease diagnosis, current treatments, and disease control status both before and after COVID-19 infection. The asthma control test was used to evaluate AS control status and was categorized as <16 (uncontrolled AS), 16‐20 (partially controlled AS), and 20‐25 (controlled AS). A low dosage of ICSs was defined as budesonide equivalence 200-400 μg, a moderate dosage as >400-800 μg, and a high dosage as >800 μg. The visual analog scale score for 3 nasal symptoms (stuffy nose, runny nose, and sneezing) was used to evaluate the AR control status. The visual analog scale score ranged from 1 to 10, with 1 indicating mild symptoms and 10 indicating severe symptoms. The third section captured COVID-19–related characteristics, such as the number of vaccination doses; the incidence, severity, and duration of COVID-19 symptoms; the recovery time to preinfection status; and the need for admission to the emergency department or hospitalization. The survey evaluated a broad spectrum of COVID-19 symptoms, including sore throat, dry cough, productive cough, chills, fever, muscle aches, dizziness, headache, diarrhea, stuffy nose, runny nose, chest tightness, fatigue, reduced or lost sense of taste or smell, difficulty breathing, chest pain, rash, heart palpitations, and joint pain. The recovery time from COVID-19 infection was determined by participants’ self-reported duration for their conditions to revert to their preinfection state, characterized by the absence of any symptoms caused by COVID-19 infection. The recovery time was categorized into the following 4 groups: within a week, 1‐2 weeks, 2‐3 weeks, and >3 weeks.

### Analysis

Statistical analysis was conducted using the SPSS software package (version 22.0; IBM Corp) or R (R Foundation for Statistical Computing). Data are expressed as means with median values and IQRs or numbers with percentages. Continuous end points were analyzed using the 2-tailed *t* test or Mann-Whitney *U* test, whereas the chi-square test was used for categorical end points to compare between groups. The primary outcomes of this study were the recovery time to preinfection status (referred to as outcome 1) and the need for admission to the emergency department or hospitalization (referred to as outcome 2). To evaluate the relative likelihood of COVID-19 outcomes, logistic regression models were used, and the odds ratios (ORs) were reported. A higher probability of the event occurring at the specified level was indicated by an OR value of >1, whereas a lower probability was indicated by an OR value of <1.

We also evaluated the effect of COVID-19 infection on AS control by using an asthma control test. A decline in AS control was defined as a transition (1) from controlled AS to partially controlled or uncontrolled AS or (2) from partially controlled AS to uncontrolled AS. Conversely, improvement in AS control referred to a transition from (1) partially controlled AS to controlled AS or (2) from uncontrolled AS to partially controlled or controlled AS. Statistical significance was set at a 2-tailed *P* value of <.05.

## Results

### Participant Allocation

In this survey, we recruited a comprehensive sample of 10,151 participants from 81 centers across China. Among these participants, 3654 (34.75%) patients and 2192 (20.85%) healthy volunteers were identified with confirmed positive test results for SARS-CoV-2 nucleic acid or antigen and were included in the analysis. No severe COVID-19 cases were reported. Conversely, 2303 (21.90%) patients and 2002 (19.04%) healthy volunteers either showed negative results for SARS-CoV-2 nucleic acid or antigen or did not undergo COVID-19 testing, resulting in their exclusion from the analysis.

Of the 3654 patients included in this study, 1939 (53.07%) were diagnosed with AR, 476 (13.03%) were diagnosed with AS, and 1239 (33.91%) were diagnosed with both conditions (AR+AS). Based on their treatment regimens, 1752 (47.95%) patients received RMT (RMT group), 1057 (28.93%) patients received AIT (Allergopharma or Alutard SQ), as an adjunct therapy to RMT (AIT+RMT group), 394 (10.78%) patients received both AIT and OMA as adjunct therapies to RMT (AIT+OMA+RMT group), and 229 (6.27%) with OMA and 222 (6.08%) with other biologics, including mepolizumab, duprizumab, and benralizumab, as adjunct therapies to RMT. Regarding the duration of AIT, 1233 (84.98%) of 1451 patients received AIT for 1‐2 years, 195 (15.82%) for 2‐3 years, and 23 (1.59%) for ≥3 years. Detailed disease profiles are presented in Table S1 in Multimedia Appendix 1.

**Figure 1.**
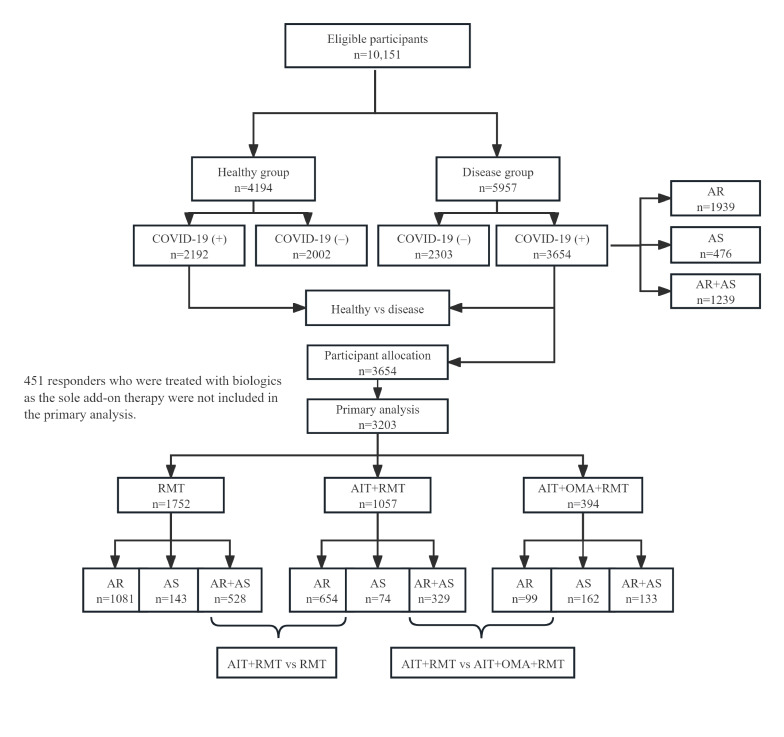
Flowchart of the design and the allocation of participants. AIT: allergen immunotherapy; AR: allergic rhinitis; AS: allergic asthma; OMA: omalizumab; RMT: routine medication treatment.

The primary analysis comprised 2 comparisons (eg, AIT+RMT vs RMT and AIT+OMA+RMT vs AIT+RMT). The objective was to ascertain whether AIT offers a protective effect against COVID-19 infection and whether the combination therapy of AIT+OMA+RMT yields superior clinical outcomes in COVID-19 infection compared to AIT+RMT. Figure 1 illustrates the flowchart depicting the study design and the allocation of participants in the primary analysis. The secondary analysis included the comparison between the disease group and the healthy group and the investigation of the impacts of OMA on COVID-19 infection. The participants receiving OMA as an adjunct therapy to RMT (OMA+RMT) only included individuals with AS; therefore, as the control group of RMT, we only included patients with AS in the RMT group (refer to Figure S1 in Multimedia Appendix 1).

### Clinical Outcomes Show No Disparities Between Disease Group and Healthy Group

Table 1 presents a comparison between the disease group, containing individuals with AR and AS, and the healthy group. Patients with AR and AS exhibited a significantly lower number of COVID-19–related symptoms (*P*<.001) and a shorter recovery period (*P*<.001) than healthy volunteers. However, there were remarkable variations in demographic characteristics, including age distribution, sex, BMI, smoking history, and COVID-19 vaccination status between the 2 groups. A separate comparison was conducted among adult participants from both groups, considering that the majority of the included healthy volunteers were adults (n=2133, 96.40%). Similarly, adult patients presented a lower incidence of symptoms than healthy volunteers. However, adults in the disease group had a higher rate of hospitalization or emergency department visits than those in the healthy group (*P*=.01).

**Table 1.** Characteristics of the study population.

	Overall			Adults		
	Disease group (n=3654)	Healthy group (n=2192)	*P* value	Disease group (n=2571)	Healthy group (n=2113)	*P* value
Sex (male), n (%)	1763 (48.25)	727 (33.17)	<.001	1028 (39.84)	683 (32.32)	<.001
BMI, median (IQR)	21.97 (19.14-24.80)	22.48 (20.20-25.05)	<.001	22.86 (20.70-25.47)	22.68 (20.40-24.67)	.79
**Age (years), n (%)**			<.001			—^a^
<18	1083 (29.64)	79 (3.60)		0 (0)	0 (0)	
≥18	2571 (70.36)	2113 (96.40)		2571 (100)	2113 (100)	
Current smoker, n (%)	152 (4.16)	147 (6.71)	<.001	152 (5.91)	147 (6.96)	.15
**COVID-19 vaccination, n (%)**			<.001^b^			<.001^b^
Not vaccinated	290 (7.94)	91 (4.15)		187 (7.27)	75 (3.55)	
One dose	160 (4.38)	40 (1.82)		108 (4.20)	33 (1.56)	
Two doses	1421 (38.89)	475 (21.67)		571 (22.21)	434 (20.54)	
Three doses	1721 (47.10)	1491 (68.02)		1645 (63.98)	1476 (69.85)	
Four doses	62 (1.70)	95 (4.33)		60 (2.33)	98 (4.64)	
**Number of COVID-19 symptoms, n (%)**			<.001^b^			.002^b^
None	194 (5.31)	92 (4.20)		110 (4.28)	81 (3.83)	
One	447 (12.23)	207 (9.44)		169 (6.58)	188 (8.90)	
Two	520 (14.23)	241 (10.99)		301 (11.71)	213 (10.08)	
Three	484 (13.24)	231 (10.54)		323 (12.56)	220 (10.41)	
Four or more	2009 (54.98)	1421 (64.82)		1668 (64.88)	1411 (66.78)	
**COVID-19 symptoms, n (%)**						
Sore throat	1347 (36.86)	1084 (49.45)	<.001	1084 (42.16)	1072 (50.73)	<.001
Dry cough	1024 (28.02)	846 (38.59)	<.001	815 (31.70)	825 (39.04)	<.001
Productive cough	1512 (41.38)	932 (42.52)	.40	1239 (48.19)	918 (43.45)	.001
Chillness	897 (24.55)	671 (30.61)	<.001	790 (30.73)	669 (31.66)	.50
Fever	2756 (75.42)	1648 (75.18)	.84	1871 (72.77)	1590 (75.25)	.06
Muscle ache	1621 (44.36)	1138 (51.92)	<.001	1404 (54.61)	1129 (53.43)	.42
Dizziness	804 (22.00)	546 (24.91)	.01	587 (22.83)	539 (25.51)	.03
Headache	1195 (32.70)	790 (36.04)	.01	938 (36.48)	780 (36.91)	.76
Diarrhea	320 (8.76)	185 (8.44)	.62	284 (11.05)	184 (8.70)	.008
Stuffy nose	1084 (29.67)	699 (31.89)	.09	857 (33.33)	691 (32.70)	.65
Runny nose	909 (24.87)	556 (25.36)	.68	711 (27.65)	548 (25.93)	.19
Chest tightness	466 (12.75)	267 (12.18)	.44	434 (16.88)	267 (12.63)	<.001
Fatigue	1302 (35.63)	1010 (46.08)	<.001	1128 (43.87)	1002 (47.42)	.02
Decreased sense of taste or smell	801 (21.92)	598 (27.28)	<.001	722 (28.08)	595 (28.16)	.95
Difficulty breathing	293 (8.02)	133 (6.07)	.005	265 (10.31)	132 (6.24)	<.001
Chest pain	156 (4.27)	97 (4.43)	.84	142 (5.52)	97 (4.59)	.15
Rash	126 (3.45)	49 (2.23)	.007	99 (3.85)	49 (2.31)	.003
Heart palpitations	272 (7.44)	202 (9.21)	.02	261 (10.15)	202 (9.56)	.50
Joint pain	834 (22.84)	565 (25.78)	.02	744 (28.94)	563 (26.64)	.08
COVID-19 infection requiring emergency visit or hospitalization, n (%)	318 (8.70)	169 (7.71)	.16	253 (9.84)	164 (7.76)	.01
**Time required to recover to the pre-infection state, n (%)**	n=3558	n=2192	<.001^b^	n=2494	n=2113	.14
Within a week	1043 (29.31)	361 (16.46)		439 (17.60)	319 (15.10)	
1‐2 weeks	791 (22.23)	478 (21.81)		527 (21.13)	451 (21.34)	
2‐3 weeks	546 (15.34)	402 (18.34)		450 (18.04)	394 (18.65)	
More than 3 weeks	1178 (33.11)	951 (43.39)		1078 (43.22)	949 (44.91)	

a Not available.

b The *P* values of chi-square analyses were validated by using Bonferroni correction.

To confirm whether AR and AS were protective factors against COVID-19 infection, we conducted univariate or multivariate logistic analysis (refer to Table S2 in Multimedia Appendix 1), adjusting for confounders, such as sex, age, BMI, smoking history, and COVID-19 vaccination. Our analysis revealed that no significant differences in the clinical outcomes of COVID-19 infection were found between the disease and healthy groups.

### Effectiveness of AIT in Improving the Clinical Characteristics of COVID-19 Infection

Table 2 illustrates that patients in the AIT+RMT group had a lower frequency of COVID-19 symptoms than those in the RMT group (*P*<.001). Furthermore, the AIT+RMT group exhibited shorter recovery periods (*P*<.001) and a reduced likelihood of hospitalization or emergency department visits compared to the RMT group (*P*=.006). However, we observed significant differences in the proportion of children and adults between the 2 groups.

**Table 2.** Comparison of demographic, clinical, and COVID-19 characteristics between patients in the AIT^a^+RMT^b^ and RMT groups.

	Total			Children			Adult		
	AIT+RMT group(n=1057)	RMT group(n=1752)	*P* value	AIT+RMT group(n=648)	RMT group(n=230)	*P* value	AIT+RMT group(n=409)	RMT group(n=1522)	*P* value
Sex (male), n (%)	613 (57.99)	683 (38.98)	<.001	450 (69.44)	149 (64.78)	.19	163 (39.85)	534 (35.09)	.08
BMI, median (IQR)	20.20 (16.30-23.72)	22.67 (20.20-25.47)	<.001	17.18 (15.15-21.10)	18.37 (15.52-22.22)	.06	23.05 (20.78-25.52)	22.89 (20.70-25.59)	.84
**Age (years), n (%)**			<.001			—^c^			—^c^
<18	648 (61.31)	230 (13.13)		648 (100)	230 (100)		0 (0)	0 (0)	
≥18	409 (38.69)	1522 (86.87)		0 (0)	0 (0)		409 (100)	1522 (100)	
Current smoker, n (%)	21 (1.99)	97 (5.54)	<.001	0 (0)	0 (0)	—^c^	21 (5.13)	97 (6.37)	.35
**Disease, n (%)**			.51			.49			.32
AR^d^	654 (61.87)	1081 (61.70)		396 (61.11)	137 (59.57)		258 (63.08)	944 (62.02)	
AS^e^	74 (7.01)	143 (8.16)		51 (7.87)	24 (10.43)		23 (5.62)	119 (7.82)	
AR and AS	329 (31.13)	528 (30.14)		201 (31.02)	69 (30)		128 (31.30)	459 (30.16)	
**Medication, n (%)**									
**Inhaled corticosteroid**	322 (30.46)	592 (33.79)	.07	175 (27.01)	62 (26.70)	.99	147 (35.94)	530 (34.82)	.68
Low dosage	197 (61.18)	236 (39.86)	<.001^f^	121 (69.14)	30 (48.39)	.006^f^	76 (51.70)	206 (38.87)	.01^f^
Moderate dosage	91 (28.26)	240 (40.54)	<.001^f^	50 (28.57)	27 (43.55)	.006^f^	41 (27.89)	213 (40.19)	.01^f^
High dosage	34 (10.56)	116 (19.59)	<.001^f^	4 (2.29)	5 (8.06)	.006^f^	30 (20.41)	111 (20.94)	.01^f^
Nasal corticosteroid	477 (45.18)	860 (49.09)	.04	313 (48.30)	112 (48.70)	.92	164 (40.10)	748 (49.14)	.001
Oral corticosteroid	0 (0)	20 (1.14)	—^c^	0 (0)	0 (0)	—^c^	0 (0)	20 (1.31)	—^c^
**Preinfection AS control condition, n (%)**	n=400	n=661	<.001^f^	n=251	n=93	.003^f^	n=149	n=568	.009^f^
Well controlled	342 (85.50)	425 (64.30)		230 (91.63)	73 (78.49)		112 (75.17)	352 (61.97)	
Not well controlled	44 (11)	167 (25.26)		16 (6.37)	17 (18.28)		28 (18.79)	150 (26.41)	
Poor controlled	14 (3.50)	69 (10.44)		5 (1.99)	3 (3.23)		9 (6.04)	66 (11.62)	
**AR symptoms score, n (%)**	n=906	n=1483		n=566	n=202		n=339	n=1281	
Stuffy nose (VAS^g^≥5)	145 (16.00)	344 (23.20)	<.001	28 (4.94)	21 (10.40)	<.001	66 (19.47)	299 (23.34)	.11
Sneezing (VAS≥5)	104 (11.48)	296 (20.00)	<.001	21 (3.71)	10 (4.95)	.87	52 (15.34)	267 (20.84)	.02
Runny nose (VAS≥5)	87 (9.60)	265 (17.87)	<.001	17 (3.00)	12 (5.94)	.47	41 (12.09)	239 (18.66)	.004
**COVID-19 vaccination, n (%)**			<.001^f^			<.001^f^			.84
Not vaccinated	67 (6.34)	132 (7.53)		45 (6.94)	36 (15.65)		22 (5.38)	96 (6.31)	
One dose	41 (3.87)	58 (3.31)		26 (4.01)	16 (6.96)		15 (3.67)	42 (2.76)	
Two doses	622 (58.85)	481 (27.45)		533 (82.25)	160 (69.57)		89 (21.76)	321 (21.09)	
Three doses	317 (30)	1044 (59.49)		44 (6.79)	18 (7.83)		273 (66.75)	1026 (67.41)	
Four doses	10 (0.95)	37 (2.11)		0 (0)	0 (0)		10 (2.44)	37 (2.43)	
**Number of COVID-19 symptoms, n (%)**			<.001^f^			.99			.15
None	65 (6.15)	62 (3.54)		53 (8.17)	19 (8.26)		12 (2.93)	43 (2.83)	
One	205 (19.39)	146 (8.33)		172 (26.54)	60 (26.09)		33 (8.07)	86 (5.65)	
Two	176 (16.65)	171 (9.76)		134 (20.68)	44 (19.13)		42 (10.27)	127 (8.34)	
Three	135 (12.77)	223 (12.73)		96 (14.81)	35 (15.22)		39 (9.53)	188 (12.35)	
Four or more	476 (45.03)	1150 (65.64)		193 (29.78)	72 (31.30)		283 (69.19)	1078 (70.83)	
**Symptoms of COVID-19 infection, n (%)**									
Sore throat	328 (31.03)	759 (43.33)	<.001	141 (21.76)	64 (27.83)	.06	187 (45.72)	695 (45.66)	.98
Cough	248 (23.46)	580 (33.11)	<.001	115 (17.75)	53 (23.04)	.08	133 (32.52)	527 (34.63)	.43
Productive cough	361 (34.15)	785 (44.81)	<.001	151 (23.30)	68 (29.57)	.06	210 (51.34)	717 (47.11)	.13
Chill	195 (18.45)	531 (30.31)	<.001	59 (9.10)	22 (9.56)	.84	136 (33.25)	509 (33.44)	.94
Fever	851 (80.51)	1343 (76.66)	.02	530 (81.79)	180 (78.26)	.24	321 (78.48)	1163 (76.41)	.38
Muscle ache	323 (30.59)	932 (53.20)	<.001	109 (16.82)	50 (21.74)	.10	214 (52.32)	882 (57.95)	.04
Dizziness	234 (22.14)	421 (24.03)	.25	136 (20.99)	37 (16.09)	.11	98 (23.96)	384 (25.23)	.60
Headache	319 (30.18)	627 (35.79)	.002	161 (24.85)	40 (17.39)	.02	158 (38.63)	587 (38.57)	.98
Diarrhea	77 (7.28)	185 (10.56)	.004	22 (3.40)	6 (2.61)	.56	55 (13.44)	179 (11.76)	.35
Stuffy nose	276 (26.11)	613 (34.99)	<.001	126 (19.44)	57 (24.78)	.09	150 (36.67)	556 (36.53)	.96
Runny nose	230 (21.75)	529 (30.19)	<.001	110 (16.97)	53 (24.78)	.04	120 (29.34)	476 (31.27)	.45
Chest tightness	80 (7.57)	289 (16.49)	<.001	13 (2.01)	12 (5.21)	.01	67 (16.38)	277 (18.19)	.40
Fatigue	284 (26.87)	758 (43.26)	<.001	102 (15.74)	38 (16.52)	.78	182 (44.50)	720 (47.31)	.31
Reduced or lost sense of taste or smell	183 (17.31)	465 (26.54)	<.001	46 (7.10)	15 (6.52)	.77	137 (33.50)	450 (29.57)	.13
Difficulty breathing	52 (4.92)	184 (10.50)	<.001	7 (1.08)	14 (6.09)	<.001	45 (11.00)	170 (11.17)	.92
Chest pain	37 (3.50)	90 (5.13)	.04	6 (0.93)	4 (1.74)	.32	31 (7.58)	86 (5.65)	.15
Rash	29 (2.74)	63 (3.59)	.22	15 (2.31)	4 (1.74)	.61	14 (3.42)	59 (3.88)	.67
Heart palpitations	43 (4.07)	176 (10.04)	<.001	3 (0.46)	7 (3.04)	.002	41 (10.02)	169 (11.10)	.53
Joint pain	161 (15.23)	486 (27.74)	<.001	48 (7.41)	18 (7.82)	.84	113 (27.62)	468 (30.75)	.22
COVID-19 infection requiring emergency visit or hospitalization, n (%)	77 (7.28)	182 (10.39)	.006	37 (5.71)	16 (6.96)	.50	40 (9.78)	166 (10.91)	.51
**Time required to recover to the preinfection state, n (%)**	n=1026	n=1709	<.001^f^	n=640	n=228	<.001^f^	n=386	n=1481	.046^f^
Within a week	441 (42.98)	298 (17.44)		381 (59.53)	106 (46.49)		68 (17.62)	194 (13.10)	
1‐2 weeks	231 (22.51)	341 (19.95)		157 (24.53)	60 (26.32)		77 (19.95)	265 (17.89)	
2‐3 weeks	134 (13.06)	313 (18.31)		55 (8.59)	25 (10.96)		74 (19.17)	282 (19.04)	
More than 3 weeks	214 (20.86)	757 (44.29)		47 (7.34)	37 (16.22)		167 (43.26)	740 (49.96)	

a AIT: allergen immunotherapy.

b RMT: routine medication treatment.

c Not available.

d AR: allergic rhinitis.

e AS: allergic asthma.

f The *P* values of chi-square analyses were validated by using Bonferroni correction.

g VAS: visual analog scale.

To address potential age-related bias, we performed subgroup comparisons between children and adult participants. Both children and adult patients in the AIT+RMT group exhibited significantly shorter recovery periods compared to those in the RMT group. Moreover, children in the AIT+RMT group also had significantly lower incidences of runny nose, chest tightness, difficulty breathing, and heart palpitations than those in the RMT group (*P*=.04, *P*=.012, *P*=.001, and *P*=.002, respectively). Additionally, both children and adult patients in the AIT+RMT group exhibited significantly shorter recovery periods than those in the RMT group. We further compared the severity and duration of symptoms between the groups (refer to Figure S2 and S3 in Multimedia Appendix 1). Children in the AIT+RMT group exhibited shorter duration of symptoms such as productive cough, dizziness, headache, fatigue, and difficulty breathing compared to those in the RMT group (*P*=.001, *P*=.01, *P*=.02, *P*=.003, and *P*=.03, respectively).

### AIT Exerted a Protective Effect in Enhancing the Clinical Outcomes of COVID-19 Infection

In this study, participants encompassed 3 disease groups (AS, AR, and AS+AR). To comprehensively evaluate the protective effect of AIT on clinical outcomes of COVID-19 infection while mitigating the effect of potential confounders within these 3 disease groups with specific clinical traits, we used 3 logistic analysis models. Model 1 (refer to Table S3 in Multimedia Appendix 1) included all patients with either AR or AS to address the common trait of AR and AS, model 2 (refer to Table S4 in Multimedia Appendix 1) comprised patients with AS to target the specific trait of AS, and model 3 (refer to Table S5 in Multimedia Appendix 1) exclusively included patients with AR to focus on the specific trait of AR. Based on the collective results of the univariate logistic analysis across these 3 models, we identified several factors associated with the clinical outcomes of COVID-19 infection in our cohort, including undergoing AIT, sex, age, BMI, COVID-19 vaccination status, AS control status, AR severity, and OMA treatment. However, we found that the daily dosage of ICSs exerted no effect on the clinical outcomes of COVID-19.

After adjusting for these confounders in the 3 multivariate logistic analysis models, AIT consistently emerged as a significant protective factor associated with a shorter recovery time (model 1: adjusted OR 0.62, 95% CI 0.52‐0.75; adjusted *P*<.001; model 2: adjusted OR 0.62, 95% CI 0.47‐0.82; adjusted *P*<.001; and model 3: adjusted OR 0.67, 95% CI 0.55‐0.82; adjusted *P*<.001) and a lower incidence of hospitalization or emergency department visits (model 1: adjusted OR 0.71, 95% CI 0.53‐0.95; adjusted *P*=.02).

### Enhanced Protective Effect of AIT+OMA Compared With AIT in COVID-19 Infection

Consistent with previous studies, our findings also demonstrated the improved antiviral effect of OMA against COVID-19 infection (refer to Table S6 in Multimedia Appendix 1). A comparison of the AIT+OMA+RMT group with the AIT+RMT group (refer to Table S7 in Multimedia Appendix 1) revealed a significantly lower incidence of COVID-19 symptoms (*P*<.001) along with a trend toward a shorter recovery time and a lower incidence of hospitalization or emergency department visits (*P*=.06 and *P*=.08, respectively).

Adults in the AIT+OMA+RMT group exhibited milder severity of sore throat, productive cough, and muscle ache than those in the AIT+RMT group (*P*=.002, *P*=.002, and *P*=.01, respectively), whereas children in the AIT+OMA+RMT group showed milder severity of dry cough and joint pain than those in the AIT+RMT group (*P*=.04 and *P*=.045, respectively; refer to Figure S4 in Multimedia Appendix 1). Furthermore, adults in the AIT+OMA+RMT group experienced a shorter duration of COVID-19 symptoms, including sore throat, dry cough, productive cough, muscle ache, and joint pain, than those in the AIT+RMT group (*P*=.006, *P*=.001, *P*<.001, *P*=.002, and *P*=.005, respectively; refer to Figure S5 in Multimedia Appendix 1).

To further investigate whether the combination of AIT and OMA exerted a superior protective effect than AIT alone, we performed additional univariate and multivariate logistic analyses (refer to Table S8 in Multimedia Appendix 1), including patients receiving either AIT or the combination of AIT and OMA as an adjunct therapy to RMT. After adjusting for confounding factors, our analyses revealed that the combination of AIT and OMA provided a significantly greater level of protection than AIT alone in terms of shorter recovery times (adjusted OR 0.51, 95% CI 0.31‐0.62; adjusted *P*<.001).

### AIT Mitigated AS Control Decline Following COVID-19 Infection

**Figure 2.**
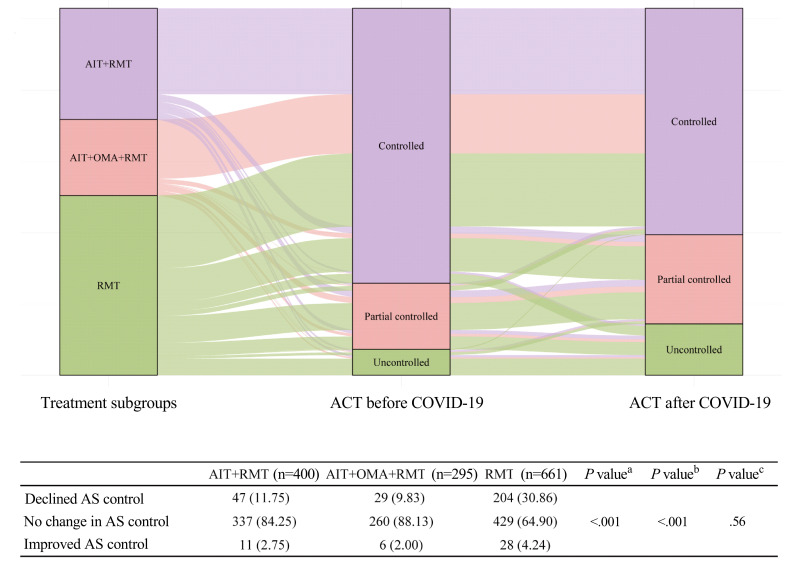
Sankey diagram illustrating the AS control transitions. A visual representation depicting the shifts in AS control states before and after the onset of COVID-19 infection. The width of the bands reflects the proportion of patients transitioning between states. ACT: asthma control test; AIT: allergen immunotherapy; AS: allergic asthma; OMA: omalizumab; RMT: routine medication treatment. ^a^AIT+RMT versus RMT; ^b^AIT+OMA+RMT versus RMT; ^c^AIT+RMT versus AIT+OMA+RMT.

Figure 2 provides a visual representation of the changes in AS control among the treatment groups before and after COVID-19 infection. We investigated the effect of COVID-19 infection on AS control in the 3 treatment groups, viz, AIT+RMT (400 patients), AIT+MA+RMT (295 patients), and RMT (661 patients). Among these groups, 11.75% (n=47) of patients in the AIT+RMT group, 9.83% (n=29) in the AIT+OMA+RMT group, and 30.86% (n=204) in the RMT group experienced a decline in AS control. The statistical analysis revealed a significantly lower prevalence of decreased AS control in both the AIT+RMT and AIT+OMA+RMT groups than in the RMT group (AIT+RMT vs RMT: *P*<.001 and AIT+OMA+RMT vs RMT: *P*<.001).

## Discussion

### Principal Findings

We conducted a multicenter survey to investigate the potential benefits of AIT in strengthening innate immunity against COVID-19 infection. Our investigation yielded significant findings, demonstrating a remarkable association between AIT and a decreased severity of COVID-19 symptoms. Moreover, individuals undergoing AIT experienced shorter recovery periods and fewer hospitalizations or emergency department visits than those undergoing RMT. Importantly, these findings remained robust even after adjusting for various covariates, including demographic characteristics, smoking history, COVID-19 vaccination status, disease severity, and disease control status. Our study also highlighted a promising trend toward enhanced protective effects of AIT+OMA against COVID-19 infection. These compelling results, derived from a large-scale survey, provide substantial empirical evidence supporting the beneficial role of AIT in the context of viral infections.

### Findings Interpretation

Allergic diseases pose an increased risk of respiratory tract infections because of the prevailing Th2 immune response [20,21]. Among therapeutic interventions, AIT stands out as the only approach that can modulate the immune response. It can alter the trajectory of allergic conditions by restoring immune homeostasis and shifting the predominance toward a more balanced Th1/Th2 response [22,23]. Notable, a nationwide population study revealed that patients with AS experience a higher risk of being prescribed with antibiotics for respiratory infections than those with nonallergic asthma. This finding suggests that treatment with AIT exerts a protective effect against such risks [24]. A recent nationwide epidemiological study found that the risk of lower respiratory tract bacterial infections in patients with AS effectively reduced through AIT [11,25]. However, the existing literature is currently limited to support the hypothesis that AIT can enhance the host defense against viral infection. Against the background of the global COVID-19 pandemic, our observations revealed a remarkable reduction in the severity of COVID-19 symptoms and improved clinical outcomes associated with AIT. This large-scale, multicenter study yields essential direct evidence clarifying the advantageous role of AIT in protecting against viral infections. Considering that the effectiveness of AIT in allergen desensitization has been completely demonstrated in previous clinical RCTs, real-world data, and systemic meta-analysis [26-29], we suggest that further RCTs are required to address the protective function of AIT in viral infection, thereby providing high-quality evidence supporting a dual effect of AIT, encompassing a reduction in allergen sensitization and an enhancement of innate immune functions.

The mechanism underlying the protective effect of AIT against viral infection remains inconclusive. The current knowledge indicates a shift toward Th1 immune profile and epithelial resolution as the underlying explanation [30-32]. A recent double-blind RCT demonstrated that AIT significantly enhanced the bronchial epithelial antiviral resistance to viral infection by elevating the levels of interferon (IFN)-β and IFN-λ produced by bronchial epithelial cells after 24 weeks of treatment [11]. Our previous study also demonstrated that AIT significantly elevated the serum levels of IFN-γ, which correlated with improvements in allergic symptoms [33]. Importantly, SARS-CoV-2 is highly susceptible to IFNs, particularly during the early stages of infection [34,35]. Increased production of IFNs may contribute to a decreased SARS-CoV-2 viral load in the airways, resulting in milder symptom severity and better clinical outcomes. Moreover, studies have shown that AIT modulates various regulatory cells involved in immune regulation, including regulatory T cells [36], regulatory B cells [37], and tolerogenic dendritic cells [38]. Consequently, these modifications of airway immunity counterbalance the Th2 immune response, priming the epithelium and submucosal environment toward a more tolerogenic state to repair the impaired epithelium.

OMA, a monoclonal antibody drug, is used to treat moderate to severe AS [39]. The combined therapy of AIT and OMA reduces the adverse effects of AIT, ameliorates allergic symptoms, and enhances disease control [40,41]. Previous reports have indicated that OMA can potentiate innate antiviral responses, mitigating the AS exacerbation induced by respiratory viral infection [18]. In this study, we also observed enhanced clinical outcomes in patients with COVID-19 who received OMA. Furthermore, we detected that a trend suggestive of a more potent protective effect of AIT+OMA than that of AIT against COVID-19 infection was observed. The mechanism underlying this increased effect can be attributed to the capability of OMA to reinstate IFN-α production from plasmacytoid dendritic cells, subsequently impeding viral replication [18,42]. Nonetheless, it is worth considering that there may exist shared pathways or interconnected mechanisms between AIT and OMA, contributing to the augmented efficacy against viral infections. Further investigations are required to investigate the clinical application of the combination therapy of AIT and OMA.

ICSs are essential in the management of AS and works by reducing airway inflammation and hyperactivity. Nevertheless, our study found no significant correlation between the dosage of ICSs and the recovery time, hospitalization rate, or emergency department visits of patients with COVID-19. These findings are consistent with recent observational studies that have demonstrated that ICS use does not affect the rates of COVID-19 infection, hospitalization rate, or mortality in patients with AR or AS [43,44]. However, evidence suggests that ICSs increase susceptibility to upper respiratory tract viral infections such as influenza and respiratory syncytial virus [45]. Mechanistically, ICSs can hinder the effectiveness of antiviral drugs, leading to delayed viral clearance in human airway epithelial cells [46]. Considering the potential protective effect of AIT against viral infection, it may be particularly recommended to consider AIT for patients with allergen-driven AR or AS who require daily corticosteroids and are susceptible to respiratory tract infection–induced exacerbation. Further studies are warranted to comprehensively investigate the enhanced antiviral functionality of AIT, ultimately facilitating its practical application in clinical settings.

### Strengths and Limitations

The strength of this study lies in the robust evidence presented, which supports the beneficial role of AIT in enhancing innate immunity against pathogens. This study was conducted as a large-scale, multicenter investigation under the challenging conditions of the COVID-19 pandemic, which adds credibility to the findings. However, several limitations need to be acknowledged. The study design did not address the mechanism underlying the enhanced protective effect of AIT against viral infection. Furthermore, the data collection relied on questionnaires, without detailed clinical measurements. Consequently, important factors, such as inflammatory indicators (eg, blood and sputum eosinophil count and fractional exhaled nitric oxide), immunoglobulin E levels, and lung function, were excluded from the analysis. Hence, the impact of these factors on the outcomes of COVID-19 infection could not be completely evaluated. Moreover, the use of other medications for COVID-19 treatment, including antitussive drugs, expectorant drugs, antipyretic drugs, and traditional Chinese medicine, may also affect the outcomes of COVID-19. Additionally, the duration of AIT might also be associated with the outcomes of COVID-19. However, the unbalanced distribution of durations of AIT in our study limited us in addressing such an issue. Further studies are required to thoroughly investigate this aspect.

### Conclusions

Our study demonstrates that individuals undergoing AIT exhibit a significant reduction in COVID-19 symptoms. AIT emerges as an independent protective factor associated with a shorter recovery time and decreased rates of hospitalization and emergency department visits in patients with COVID-19. Importantly, our investigation provides clinical evidence that substantiates the potential augmentation of the antiviral response facilitated by AIT. Further scientific inquiry in this domain holds promise for clarifying the underlying mechanisms and providing valuable insights for future therapeutic interventions.

## Supplementary material

10.2196/50846 Multimedia Appendix 1 Supplementary tables and figures with further data on disease profiles, COVID-19 severity and duration, univariate and multivariate logistic analyses, and group comparisons.

10.2196/50846 Multimedia Appendix 2 Detailed information of contributors to our work.

## Abbreviations

AIT: allergen immunotherapy
AR: allergic rhinitis
AS: allergic asthma
ICS: inhaled corticosteroid
IFN: interferon
OMA: omalizumab
OR: odds ratio
RCT: randomized controlled trial
RMT: routine medication treatment
