# Supplementary material for: Protective Effect of Allergen Immunotherapy in Patients With Allergic Rhinitis and Asthma Against COVID-19 Infection: Observational, Nationwide, and Multicenter Study
Source: JMIR Public Health Surveill. 2024 Oct 16;10:e50846. doi: 10.2196/50846 (PMC11498206; doi:10.2196/50846)
Supplement: Multimedia Appendix 1 [file publichealth-v10-e50846-s001.docx]

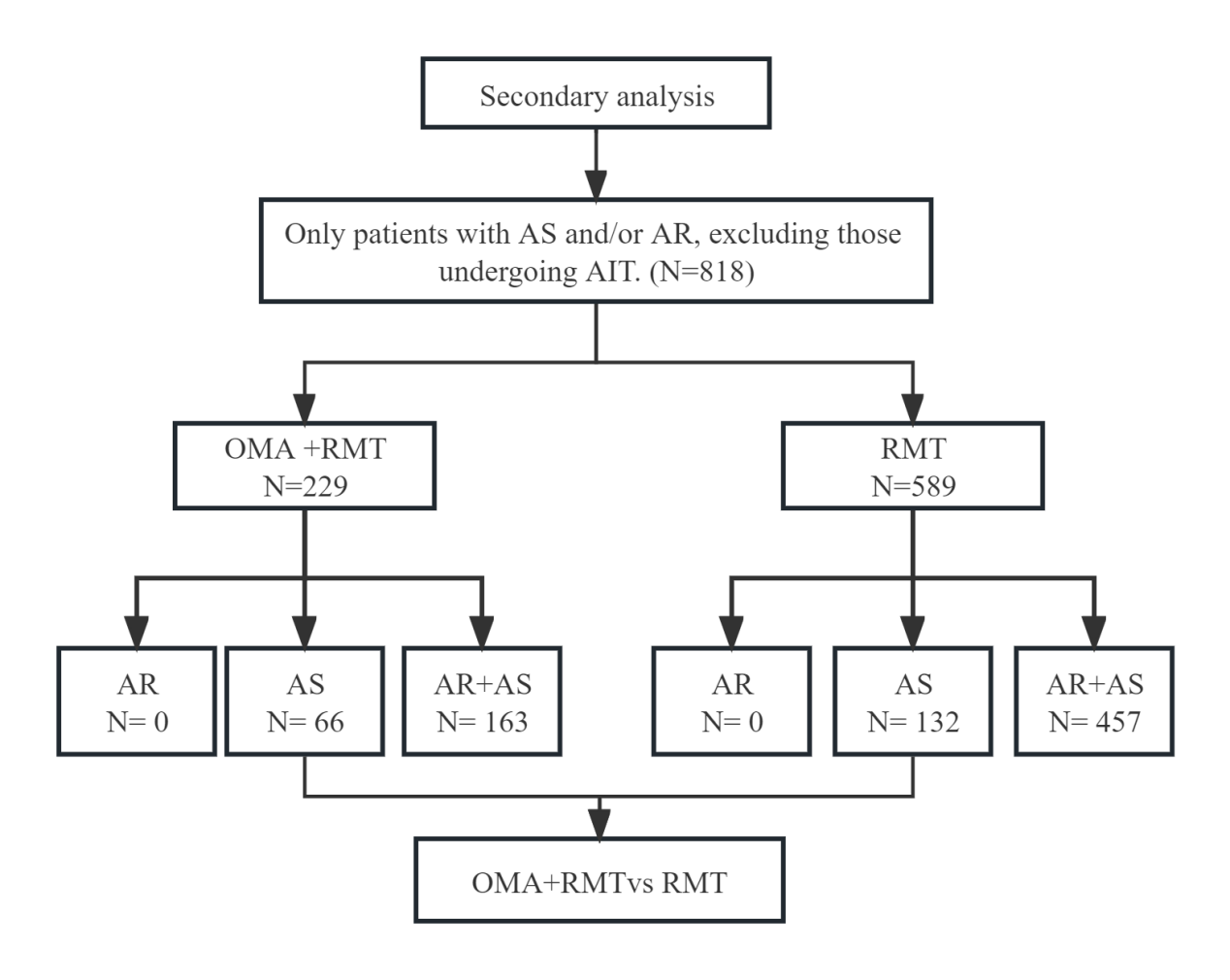


Figure S1. illustrates the flowchart depicting the secondary analysis and the allocation of participants.

Figure S2. Comparison of COVID-19 related symptoms severity between AIT+RMT and RMT groups (adult and children subgroups). Abbreviations: AIT, allergen immunotherapy; OMA, omalizumab; RMT, routine medication treatment; VAS, visual analog scale. The symbols * indicates that the comparison was not performed due to a limited number of subjects (number ≤ 10).

Figure S3. Comparison of COVID-19 related symptoms duration between AIT+RMT and RMT groups (adult and children subgroups). Abbreviations: AIT, allergen immunotherapy; OMA, omalizumab; RMT, routine medication treatment; The symbols * indicates that the comparison was not performed due to a limited number of subjects (N<10).

Figure S4. Comparison of COVID-19 related symptoms severity between AIT+RMT and AIT+OMA+RMT groups (adult and children subgroups). Abbreviations: AIT, allergen immunotherapy; OMA, omalizumab; RMT, routine medication treatment; VAS, visual Analog Scale. The symbols * indicates that the comparison was not performed due to a limited number of subjects (number ≤ 10).

Figure S5. Comparison of COVID-19 related symptoms duration between AIT+RMT and AIT+OMA+RMT groups (adult and children subgroups). Abbreviations: AIT, allergen immunotherapy; OMA, omalizumab; RMT, routine medication treatment; The symbols * indicates that the comparison was not performed due to a limited number of subjects (N≤10).

| Table S1. Disease profile in the disease group. | |
| --- | --- |
|  | Disease group (N = 3654) |
| **Disease^a^** |  |
| AR^d^ | 1939 (53.07) |
| AS^e^ | 476 (13.03) |
| AR and AS | 1239 (33.91) |
| **Treatment regimens^a^** |  |
| RMT | 1752 (47.95) |
| AIT+RMT | 1057 (28.93) |
| AIT+OMA+RMT | 394 (10.78) |
| OMA+RMT | 229 (6.27) |
| Other biologies+RMT | 222 (6.08) |
| **The duration of AIT^a^** | N=1451 |
| 1-2y | 1233 (84.98) |
| 2-3y | 195 (15.82) |
| ≥3y | 23 (1.59) |

^a^AR: allergic rhinitis.

^b^AS: allergic asthma.

^c^RMT: routine medication treatment

^d^AIT: allergen immunotherapy

^e^OMA: omalizumab

| Table S2. Univariate and multivariate logistic analysis to identify the factors contributing to COVID-19 outcomes for all included subjects. | | | | | | | | | | | | | | | | | |
| --- | --- | --- | --- | --- | --- | --- | --- | --- | --- | --- | --- | --- | --- | --- | --- | --- | --- |
|  | To identify the factors contributing to RT^a^ < 3w (outcome 1) | | | | | | | |  | To identify the factors contributing to Non-HO^b^ or ER^c^ (outcome 2) | | | | | | | |
|  | Univariate logistic analysis | | | | Multivariate logistic analysis | | | |  | Univariate logistic analysis | | | | Multivariate logistic analysis | | | |
| Variables | RT < 3w  (N, %) | RT ≥ 3w  (N, %) | OR  (95%CI) | P- value | RT < 3w  (N, %) | RT ≥ 3w  (N, %) | Adjusted-OR  (95%CI) | P- value |  | Non-HO or ER  (N, %) | HO or ER  (N, %) | OR  (95%CI) | P-value | Non-HO or ER  (N, %) | HO or ER  (N, %) | Adjusted-OR  (95%CI) | P- value |
| Age (years) | N = 3835 | N=2277 |  |  | N = 3598 | N = 2119 |  |  |  | N = 5719 | N = 545 |  |  | N = 5682 | N = 540 |  |  |
| <18 | 1069 (27.9%) | 107  (4.7%) | Reference |  | 1034 (28.7%) | 100  (4.7%) | Reference |  |  | 1126  (19.7%) | 73  (13.4%) | Reference |  | 1116 (19.6%) | 73  (13.5%) | Reference |  |
| >=18 | 2766 (72.1%) | 2170 (95.3%) | 7.84 (6.38-9.64) | <.001 | 2564 (71.3%) | 2019 (95.3%) | 5.69 (4.46-7.26) | <.001 |  | 4593  (80.3%) | 472 (86.6%) | 1.59 (1.23-2.05) | <.001 | 4566 (80.4%) | 467 (86.5%) | 1.39 (1.03-1.87) | .032 |
| Gender | N = 3835 | N = 2276 |  |  | N = 3598 | N = 2119 |  |  |  | N = 5716 | N = 545 |  |  |  |  |  |  |
| Female | 1960 (51.1%) | 1529 (67.2%) | Reference |  | 1849 (51.4%) | 1439 (67.9%) | Reference |  |  | 3264  (57.1%) | 310 (56.9%) | Reference |  |  |  |  |  |
| Male | 1875 (48.9%) | 747  (32.8%) | 0.51  (0.46-0.57) | <.001 | 1749 (48.6%) | 680  (32.1%) | 0.58 (0.51-0.66) | <.001 |  | 2452  (42.9%) | 235 (43.1%) | 1.01 (0.85-1.21) | .920 |  |  |  |  |
| BMI^d^ | N = 3809 | N = 2263 |  |  | N = 3598 | N = 2119 |  |  |  | N = 5682 | N = 540 |  |  | N = 5682 | N = 540 |  |  |
| 18–23.5 | 2154 (56.6%) | 1439 (63.6%) | Reference |  | 2025 (56.3%) | 1355 (63.9%) | Reference |  |  | 3354  (59%) | 326 (60.4%) | Reference |  | 3354  (59.0%) | 326 (60.4%) | Reference |  |
| <18 | 864  (22.7%) | 184  (8.1%) | 0.32 (0.27-0.38) | <.001 | 821  (22.8%) | 172  (8.1%) | 0.70 (0.57-0.87) | <.001 |  | 1000  (17.6%) | 68  (12.6%) | 0.7  (0.53-0.92) | .010 | 1000 (17.6%) | 68  (12.6%) | 0.84 (0.62-1.15) | .271 |
| >23.5 | 791  (20.8%) | 640  (28.3%) | 1.21 (1.07-1.37) | .002 | 752  (20.9%) | 592  (27.9%) | 1.28 (1.12-1.46) | <.001 |  | 1328  (23.4%) | 146  (27%) | 1.13 (0.92-1.39) | .239 | 1328 (23.4%) | 146  (27%) | 1.09 (0.88-1.34) | .432 |
| Current smoker | N = 3835 | N = 2277 |  |  | N = 3598 | N = 2119 |  |  |  | N = 5719 | N = 545 |  |  | N = 5682 | N = 540 |  |  |
| No | 3394 (88.5%) | 1956 (85.9%) | Reference |  | 3193 (88.7%) | 1836 (86.6%) | Reference |  |  | 5034  (88%) | 446 (81.8%) | Reference |  | 5001  (88.0%) | 442 (81.9%) | Reference |  |
| Yes | 441  (11.5%) | 321  (14.1%) | 1.26 (1.08-1.47) | .003 | 405  (11.3%) | 283  (13.4%) | 1.16 (0.97-1.39) | .114 |  | 685  (12%) | 99  (18.2%) | 1.63 (1.29-2.06) | <.001 | 681  (12.0%) | 98  (18.1%) | 1.47 (1.16-1.87) | .002 |
| COVID-19 Vaccination | N = 3835 | N = 2277 |  |  | N = 3598 | N = 2119 |  |  |  | N = 5719 | N = 545 |  |  | N = 5682 | N = 540 |  |  |
| Unvaccinated | 239  (6.2%) | 174  (7.6%) | Reference |  | 215  (6.0%) | 154  (7.3%) | Reference |  |  | 368  (6.4%) | 57  (10.5%) | Reference |  | 363  (6.4%) | 56  (10.4%) | Reference |  |
| One dose | 141  (3.7%) | 72  (3.2%) | 0.7  (0.50-0.99) | .044 | 132  (3.7%) | 63  (3.0%) | 0.65 (0.44-0.96) | .030 |  | 192  (3.4%) | 28  (5.1%) | 0.94 (0.58-1.53) | .807 | 189  (3.3%) | 28  (5.2%) | 0.96 (0.59-1.57) | .870 |
| Two or more dose | 3455 (90.1%) | 2031 (89.2%) | 0.81 (0.66-0.99) | .039 | 3251 (90.4%) | 1902 (89.8%) | 0.64 (0.51-0.82) | <.001 |  | 5159  (90.2%) | 460 (84.4%) | 0.58 (0.43-0.77) | <.001 | 5130 (90.3%) | 456 (84.4%) | 0.56 (0.41-0.75) | <.001 |
| Groups | N = 3621 | N = 2129 |  |  | N = 3598 | N = 2119 |  |  |  | N = 5359 | N = 487 |  |  |  |  |  |  |
| Health | 1241 (34.3%) | 951  (44.7%) | Reference |  | 1231 (34.2%) | 949  (44.8%) | Reference |  |  | 2023  (37.7%) | 169 (34.7%) | Reference |  |  |  |  |  |
| Disease | 2380 (65.7%) | 1178 (55.3%) | 0.65 (0.58-0.72) | <.001 | 2367 (65.8%) | 1170 (55.2%) | 0.95 (0.85-1.07) | .403 |  | 3336  (62.3%) | 318 (65.3%) | 1.14 (0.94-1.39) | .184 |  |  |  |  |

^a^RT: recovery time.

^b^HO: hospitalization.

^c^ER: emergency department visit.

^d^BMI: body mass index.

| Table S3. Univariate and multivariate logistic analysis to identify the factors contributing to COVID-19 outcomes for subjects in the disease group. | | | | | | | | | | | | | | | | | |
| --- | --- | --- | --- | --- | --- | --- | --- | --- | --- | --- | --- | --- | --- | --- | --- | --- | --- |
|  | To identify the factors contributing to RT^a^ < 3w (outcome 1) | | | | | | | |  | To identify the factors contributing to Non-HO^b^ or ER^c^ (outcome 2) | | | | | | | |
|  | Univariate logistic analysis | | | | Multivariate logistic analysis | | | |  | Univariate logistic analysis | | | | Multivariate logistic analysis | | | |
| Variables | RT < 3w  (N, %) | RT ≥ 3w  (N, %) | OR  (95%CI) | P-value | RT < 3w  (N,%) | RT ≥ 3w  (N,%) | Adjusted-OR  (95%CI) | P- value |  | Non-HO or ER  (N,%) | HO or ER  (N,%) | OR  (95%CI) | p value | Non-HO or ER  (N,%) | HO or ER  (N,%) | Adjusted-OR  (95%CI) | P- value |
| Age (years) | N = 2448 | N = 1170 |  |  | N = 2291 | N = 1116 |  |  |  | N = 3301 | N = 317 |  |  | N = 3121 | N = 302 |  |  |
| <18 | 955  (39.0%) | 92  (7.9%) | Reference |  | 891  (38.9%) | 89  (8.0%) | Reference |  |  | 983 (29.8%) | 64 (20.2%) | Reference |  | 923  (29.6%) | 62  (20.5%) |  |  |
| >=18 | 1493 (61.0%) | 1078 (92.1%) | 7.5  (5.97-9.41) | <.001 | 1400 (61.1%) | 1027  (92.0%) | 4.59 (3.45-6.10) | <.001 |  | 2318 (70.2%) | 253 (79.8%) | 1.68 (1.26-2.23) | <.001 | 2198 (70.4%) | 240 (79.5%) | 1.28 (0.92-1.79) | .145 |
| Gender | N = 2447 | N = 1170 |  |  | N = 2291 | N = 1116 |  |  |  | N = 3300 | N = 317 |  |  |  |  |  |  |
| Female | 1113 (45.5%) | 763  (65.2%) | Reference |  | 1046 (45.7%) | 735 (65.9%) | Reference |  |  | 1697 (51.4%) | 179 (56.5%) | Reference |  |  |  |  |  |
| Male | 1334 (54.5%) | 407  (34.8%) | 0.45 (0.39-0.51) | <.001 | 1245 (54.3%) | 381 (34.1%) | 0.58 (0.49-0.70) | <.001 |  | 1603 (48.6%) | 138 (43.5%) | 0.82 (0.65-1.03) | .086 |  |  |  |  |
| BMI^d^ | N = 2435 | N = 1164 |  |  | N = 2291 | N = 1116 |  |  |  | N = 3284 | N = 315 |  |  |  |  |  |  |
| 18–23.5 | 1307 (53.7%) | 708  (60.8%) | Reference |  | 1231 (53.7%) | 678 (60.8%) | Reference |  |  | 1840  (56%) | 175 (55.6%) | Reference |  |  |  |  |  |
| <18 | 645  (26.5%) | 108  (9.3%) | 0.31 (0.25-0.39) | <.001 | 616  (26.9%) | 107  (9.6%) | 0.83 (0.63-1.09) | .175 |  | 704 (21.4%) | 49 (15.6%) | 0.73 (0.53-1.02) | .062 |  |  |  |  |
| >23.5 | 483  (19.8%) | 348  (29.9%) | 1.33 (1.13-1.57) | <.001 | 444  (19.4%) | 331 (29.7%) | 1.34 (1.12-1.61) | .002 |  | 740 (22.5%) | 91 (28.9%) | 1.29 (0.99-1.69) | .060 |  |  |  |  |
| Current smoker | N = 2448 | N = 1170 |  |  | N = 2291 | N = 1116 |  |  |  | N = 3301 | N = 317 |  |  | N = 3121 | N = 302 |  |  |
| No | 2163 (88.4%) | 993  (84.9%) | Reference |  | 2034 (88.8%) | 955 (85.6%) | Reference |  |  | 2902 (87.9%) | 254 (80.1%) | Reference |  | 2758 (88.4%) | 245 (81.1%) |  |  |
| Yes | 285  (11.6%) | 177  (15.1%) | 1.35 (1.11-1.66) | .003 | 257  (11.2%) | 161 (14.4%) | 1.13 (0.89-1.44) | .309 |  | 399  (12.1%) | 63 (19.9%) | 1.8 (1.34-2.42) | <.001 | 363  (11.6%) | 57  (18.9%) | 1.62 (1.18-2.23) | .003 |
| COVID-19 vaccination | N = 2448 | N = 1170 |  |  | N = 2291 | N = 1116 |  |  |  | N = 3301 | N = 317 |  |  |  |  |  |  |
| Unvaccinated | 160  (6.5%) | 111  (9.5%) | Reference |  | 136  (5.9%) | 105  (9.4%) | Reference |  |  | 241  (7.3%) | 30  (9.5%) | Reference |  |  |  |  |  |
| One dose | 116  (4.7%) | 39  (3.3%) | 0.48 (0.31-0.75) | <.001 | 110  (4.8%) | 37  (3.3%) | 0.49 (0.30-0.80) | .005 |  | 137  (4.2%) | 18  (5.7%) | 1.06 (0.57-1.96) | .865 |  |  |  |  |
| Two or more doses | 2172 (88.7%) | 1020 (87.2%) | 0.68 (0.53-0.87) | .002 | 2045 (89.3%) | 974 (87.3%) | 0.57 (0.42-0.77) | <.001 |  | 2923 (88.5%) | 269 (84.9%) | 0.74 (0.50-1.10) | .138 |  |  |  |  |
| Diseases | N = 2448 | N = 1170 |  |  |  |  |  |  |  | N = 3301 | N = 317 |  |  |  |  |  |  |
| AR+AS | 828  (33.8%) | 402  (34.4%) | Reference |  |  |  |  |  |  | 1110 (33.6%) | 120 (37.9%) | Reference |  |  |  |  |  |
| AS | 343  (14.0%) | 133  (11.4%) | 0.80 (0.63-1.01) | .059 |  |  |  |  |  | 433 (13.1%) | 43 (13.6%) | 0.92 (0.64-1.32) | .649 |  |  |  |  |
| AR | 1277 (52.2%) | 635  (54.3%) | 1.02 (0.88-1.19) | .758 |  |  |  |  |  | 1758 (53.3%) | 154 (48.6%) | 0.81 (0.63-1.04) | .099 |  |  |  |  |
| AIT | N = 2301 | N = 1122 |  |  | N = 2291 | N = 1116 |  |  |  | N = 3121 | N = 302 |  |  | N = 3121 | N = 302 |  |  |
| No | 1123 (48.8%) | 849  (75.7%) | Reference |  | 1117 (48.8%) | 844 (75.6%) | Reference |  |  | 1765 (56.6%) | 207 (68.5%) | Reference |  | 1765 (56.6%) | 207 (68.5%) |  |  |
| Yes | 1178 (51.2%) | 273  (24.3%) | 0.31 (0.26-0.36) | <.001 | 1174 (51.2%) | 272 (24.4%) | 0.62 (0.52-0.75) | <.001 |  | 1356 (43.4%) | 95 (31.5%) | 0.6 (0.46-0.77) | <.001 | 1356 (43.4%) | 95  (31.5%) | 0.71 (0.53-0.95) | .021 |
| OMA | N = 2448 | N = 1170 |  |  | N = 2291 | N = 1116 |  |  |  | N = 3301 | N = 317 |  |  | N = 3121 | N = 302 |  |  |
| No | 1957 (79.9%) | 1011 (86.4%) | Reference |  | 1803 (78.7%) | 957 (85.8%) | Reference |  |  | 2695 (81.6%) | 273 (86.1%) | Reference |  | 2515 (80.6%) | 258 (85.4%) |  |  |
| Yes | 491  (20.1%) | 159  (13.6%) | 0.63 (0.52-0.76) | <.001 | 488  (21.3%) | 159 (14.2%) | 0.63 (0.51-0.78) | <.001 |  | 606  (18.4%) | 44  (13.9%) | 0.72 (0.52-1.00) | .048 | 606  (19.4%) | 44  (14.6%) | 0.73 (0.52-1.03) | .071 |

^a^RT: recovery time.

^b^HO: hospitalization.

^c^ER: emergency department visit.

^d^BMI: body mass index.

| Table S4. Univariate and multivariate logistic analysis to identify the factors contributing to COVID-19 outcomes for subjects with AS and/or AR. | | | | | | | | | | | | | | | | | |
| --- | --- | --- | --- | --- | --- | --- | --- | --- | --- | --- | --- | --- | --- | --- | --- | --- | --- |
|  | To identify the factors contributing to RT^a^ < 3w (outcome 1) | | | | | | | |  | To identify the factors contributing to Non-HO^b^ or ER^c^ (outcome 2) | | | | | | | |
|  | Univariate logistic analysis | | | | Multivariate logistic analysis | | | |  | Univariate logistic analysis | | | | Multivariate logistic analysis | | | |
| Variables | RT < 3w  (N, %) | RT ≥ 3w  (N, %) | OR  (95%CI) | P- value | RT < 3w  (N, %) | RT ≥ 3w  (N, %) | Adjusted-OR  (95%CI) | P- value |  | Non-HO or ER  (N, %) | HO or ER  (N, %) | OR  (95%CI) | P- value | Non-HO or ER  (N, %) | HO or ER  (N, %) | Adjusted-OR  (95%CI) | P- value |
| Age (years) | N = 1171 | N = 535 |  |  | N = 1046 | N = 481 |  |  |  | N = 1543 | N = 163 |  |  | N = 1387 | N = 148 |  |  |
| <18 | 409  (34.9%) | 41  (7.7%) | Reference |  | 377  (36.0%) | 38  (7.9%) | Reference |  |  | 418  (27.1%) | 32  (19.6%) | Reference |  | 388  (28.0%) | 30  (20.3%) | Reference |  |
| >=18 | 762  (65.1%) | 494 (92.3%) | 6.47 (4.60-9.09) | <.001 | 669  (64.0%) | 443 (92.1%) | 4.28 (2.75-6.65) | <.001 |  | 1125  (72.9%) | 131 (80.4%) | 1.52 (1.02-2.27) | .041 | 999  (72.0%) | 118  (79.7%) | 1.09 (0.68-1.77) | .713 |
| Gender | N = 1170 | N = 535 |  |  | N = 1046 | N = 481 |  |  |  | N = 1542 | N = 163 |  |  | N = 1387 | N = 148 |  |  |
| Female | 512  (43.8%) | 353  (66%) | Reference |  | 466 (44.6%) | 320 (66.5%) | Reference |  |  | 767  (49.7%) | 98  (60.1%) | Reference |  | 702  (50.6%) | 88  (59.5%) | Reference |  |
| Male | 658  (56.2%) | 182  (34.0%) | 0.40 (0.32-0.50) | <.001 | 580 (55.4%) | 161 (33.5%) | 0.54 (0.42-0.71) | <.001 |  | 775  (50.3%) | 65  (39.9%) | 0.66 (0.47-0.91) | .012 | 685  (49.4%) | 60  (40.5%) | 0.86 (0.60-1.23,) | .406 |
| BMI^d^ | N = 1164 | N = 532 |  |  | N = 1046 | N = 481 |  |  |  | N = 1534 | N = 162 |  |  |  |  |  |  |
| 18-23.5 | 640  (55%) | 300 (56.4%) | Reference |  | 565  (54.0%) | 270 (56.1%) | Reference |  |  | 851  (55.5%) | 89  (54.9%) | Reference |  |  |  |  |  |
| <18 | 277  (23.8%) | 50  (9.4%) | 0.39 (0.28-0.54) | <.001 | 259  (24.8%) | 46  (9.6%) | 0.99 (0.65-1.52) | .970 |  | 300  (19.6%) | 27  (16.7%) | 0.86 (0.55-1.35) | .513 |  |  |  |  |
| >23.5 | 247  (21.2%) | 182 (34.2%) | 1.57 (1.24-1.99) | <.001 | 222  (21.2%) | 165 (34.3%) | 1.42 (1.08-1.86) | .011 |  | 383  (25.0%) | 46  (28.4%) | 1.15 (0.79-1.67) | .470 |  |  |  |  |
| Current smoker | N = 1171 | N = 535 |  |  | N = 1046 | N = 481 |  |  |  | N = 1543 | N = 163 |  |  |  |  |  |  |
| No | 1007  (86.0%) | 440 (82.2%) | Reference |  | 907  (86.7%) | 395 (82.1%) | Reference |  |  | 1317 (85.4%) | 130 (79.8%) | Reference |  |  |  |  |  |
| Yes | 164  (14.0%) | 95  (17.8%) | 1.33 (1.01-1.75) | .046 | 139  (13.3%) | 86  (17.9%) | 1.23 (0.87-1.74) | .238 |  | 226  (14.6%) | 33  (20.2%) | 1.48 (0.98-2.22) | .059 |  |  |  |  |
| COVID-19 vaccination | N = 1171 | N = 535 |  |  | N = 1046 | N = 481 |  |  |  | N = 1543 | N = 163 |  |  |  |  |  |  |
| Unvaccinated | 88  (7.5%) | 86  (16.1%) | Reference |  | 71  (6.8%) | 78  (16.2%) | Reference |  |  | 151  (9.8%) | 23  (14.1%) | Reference |  |  |  |  |  |
| One dose | 71  (6.1%) | 18  (3.4%) | 0.26 (0.14-0.47) | <.001 | 65  (6.2%) | 16  (3.3%) | 0.25 (0.13-0.50) | <.001 |  | 78  (5.1%) | 11  (6.7%) | 0.93 (0.43-2.00) | .844 |  |  |  |  |
| Two or more dose | 1012 (86.4%) | 431 (80.6%) | 0.44 (0.32-0.60) | <.001 | 910  (87.0%) | 387 (80.5%) | 0.38 (0.26-0.55) | <.001 |  | 1314 (85.2%) | 129 (79.1%) | 0.64 (0.40-1.04) | .070 |  |  |  |  |
| Diseases | N = 1171 | N = 535 |  |  |  |  |  |  |  | N = 1543 | N = 163 |  |  |  |  |  |  |
| AR+AS | 828  (70.7%) | 402 (75.1%) | Reference |  |  |  |  |  |  | 1110  (71.9%) | 120 (73.6%) | Reference |  |  |  |  |  |
| AS | 343  (29.3%) | 133 (24.9%) | 0.80 (0.63-1.01) | .059 |  |  |  |  |  | 433  (28.1%) | 43  (26.4%) | 0.92 (0.64-1.32) | .649 |  |  |  |  |
| The dosage of daily ICS^e^ | N = 1017 | N = 478 |  |  |  |  |  |  |  | N = 1353 | N = 142 |  |  |  |  |  |  |
| Low | 397  (39.0%) | 187 (39.1%) | Reference |  |  |  |  |  |  | 526  (38.9%) | 58  (40.8%) | Reference |  |  |  |  |  |
| Moderate | 426  (41.9%) | 186 (38.9%) | 0.93 (0.73-1.18) | .543 |  |  |  |  |  | 563  (41.6%) | 49  (34.5%) | 0.79 (0.53-1.18) | .244 |  |  |  |  |
| High | 194  (19.1%) | 105 (22%) | 1.15 (0.86-1.54) | .355 |  |  |  |  |  | 264  (19.5%) | 35  (24.6%) | 1.2 (0.77-1.88) | .417 |  |  |  |  |
| ACT^f^ score | N = 1131 | N = 514 |  |  | N = 1046 | N = 481 |  |  |  | N = 1488 | N = 157 |  |  | N = 1387 | N = 148 |  |  |
| >=21 | 891  (78.8%) | 314 (61.1%) | Reference |  | 828  (79.2%) | 296 (61.5%) | Reference |  |  | 1116  (75.0%) | 89  (56.7%) | Reference |  | 1044  (75.3%) | 85  (57.4%) | Reference |  |
| 16-20 | 190  (16.8%) | 137 (26.7%) | 2.05 (1.59-2.64) | <.001 | 171  (16.3%) | 127 (26.4%) | 1.40 (1.05-1.86) | .023 |  | 282  (19.0%) | 45  (28.7%) | 2 (1.37-2.93) | <.001 | 261  (18.8%) | 40  (27.0%) | 1.67 (1.10-2.51 | .015 |
| <=15 | 50  (4.4%) | 63  (12.3%) | 3.58 (2.41-5.30) | <.001 | 47  (4.5%) | 58  (12.1%) | 2.20 (1.42-3.41) | <.001 |  | 90  (6.0%) | 23  (14.6%) | 3.2 (1.93-5.32) | <.001 | 82  (5.9%) | 23  (15.5%) | 2.82 (1.66-4.78) | <.001 |
| AIT | N = 1086 | N = 503 |  |  | N = 1046 | N = 481 |  |  |  | N = 1436 | N = 153 |  |  | N = 1387 | N = 148 |  |  |
| No | 513  (47.2%) | 378 (75.1%) | Reference |  | 503  (48.1%) | 364 (75.7%) | Reference |  |  | 784  (54.6%) | 107 (69.9%) | Reference |  | 767  (55.3%) | 106  (71.6%) | Reference |  |
| Yes | 573  (52.8%) | 125 (24.9%) | 0.30 (0.23-0.37) | <.001 | 543  (51.9%) | 117 (24.3%) | 0.62 (0.47-0.82) | <.001 |  | 652  (45.4%) | 46  (30.1%) | 0.52 (0.36-0.74) | <.001 | 620  (44.7%) | 42  (28.4%) | 0.65 (0.43-1.00) | .051 |
| OMA | N = 1171 | N = 535 |  |  | N = 1046 | N = 481 |  |  |  | N = 1543 | N = 163 |  |  | N = 1387 | N = 148 |  |  |
| No | 393  (33.6%) | 131 (24.5%) | Reference |  | 680  (65%) | 361 (75.1%) | Reference |  |  | 1053 (68.2%) | 129 (79.1%) | Reference |  | 930  (67.1%) | 117  (79.1%) | Reference |  |
| Yes | 778  (66.4%) | 404 (75.5% ) | 0.64 (0.51-0.81) | <.001 | 366  (35.0%) | 120 (24.9%) | 0.60 (0.46-0.80) | <.001 |  | 490  (31.8%) | 34  (20.9%) | 0.57 (0.38-0.84) | .005 | 457  (32.9%) | 31  (20.9%) | 0.60 (0.39-0.92) | .020 |

^a^RT: recovery time.

^b^HO: hospitalization.

^c^ER: emergency department visit.

^d^BMI: body mass index.

^e^ICS: inhaled corticosteroid.

^f^ACT: asthma control test.

| Table S5. Univariate and multivariate logistic analysis to identify the factors contributing to COVID-19 outcomes for subjects with AR and/or AS. | | | | | | | | | | | | | | | | | |
| --- | --- | --- | --- | --- | --- | --- | --- | --- | --- | --- | --- | --- | --- | --- | --- | --- | --- |
|  | To identify the factors contributing to RT^a^ < 3w (outcome 1) | | | | | | | |  | To identify the factors contributing to Non-HO^b^ or ER^c^ (outcome 2) | | | | | | | |
|  | Univariate logistic analysis | | | | Multivariate logistic analysis | | | |  | Univariate logistic analysis | | | | Multivariate logistic analysis | | | |
| Variables | RT < 3w  (N, %) | RT ≥ 3w  (N, %) | OR  (95%CI) | P- value | RT < 3w  (N, %) | RT ≥ 3w  (N, %) | Adjusted-OR  (95%CI) | P-value |  | Non-HO or ER  (N, %) | HO or ER  (N, %) | OR  (95%CI) | P- value | Non-HO or ER  (N, %) | HO or ER  (N, %) | Adjusted-OR  (95%CI) | P- value |
| Age (years) | N = 2105 | N = 1037 |  |  | N = 1781 | N = 926 |  |  |  | N = 2868 | N = 274 |  |  | N = 2471 | N = 236 |  |  |
| <18 | 865 (41.1%) | 86  (8.3%) | Reference |  | 763  (42.8%) | 82  (8.9%) | Reference |  |  | 894  (31.2%) | 57 (20.8%) | Reference |  | 797 (32.3%) | 48 (20.3%) | Reference |  |
| >=18 | 1240 (58.9%) | 951 (91.7%) | 7.71 (6.09-9.78) | <.001 | 1018 (57.2%) | 844 (91.1%) | 4.94 (3.63-6.72) | <.001 |  | 1974 (68.8%) | 217 (79.2%) | 1.72 (1.27-2.33) | <.001 | 1674 (67.7%) | 188 (79.7%) | 1.45 (0.93-2.26) | .105 |
| Gender | N = 2104 | N = 1037 |  |  | N = 1781 | N = 926 |  |  |  | N = 2867 | N = 274 |  |  | N = 2471 | N = 236 |  |  |
| Female | 988  (47.0%) | 683 (65.9%) | Reference |  | 835  (46.9%) | 610 (65.9%) | Reference |  |  | 1519  (53.0%) | 152 (55.5%) | Reference |  | 1318 (53.3%) | 127 (53.8%) | Reference |  |
| Male | 1116  (53.0%) | 354 (34.1%) | 0.46 (0.39-0.54) | <.001 | 946  (53.1%) | 316 (34.1%) | 0.62 (0.51-0.76) | <.001 |  | 1348  (47%) | 122 (44.5%) | 0.9 (0.7-1.16) | .430 | 1153 (46.7%) | 109 (46.2%) | 0.96 (0.71-1.31) | .801 |
| BMI^d^ | N = 2094 | N = 1033 |  |  | N = 1781 | N = 926 |  |  |  | N = 2854 | N = 273 |  |  | N = 2471 | N = 236 |  |  |
| 18-23.5 | 1090 (52.1%) | 644 (62.3%) | Reference |  | 929  (52.2%) | 569 (61.4%) | Reference |  |  | 1577 (55.3%) | 157 (57.5%) | Reference |  | 1360  (55.0%) | 138 (58.5%) | Reference |  |
| <18 | 573 (27.4%) | 97  (9.4%) | 0.29 (0.23-0.36) | <.001 | 505  (28.4%) | 93  (10%) | 0.88 (0.65-1.18) | .387 |  | 627  (22.0%) | 43 (15.8%) | 0.69 (0.49-0.98) | .037 | 563 (22.8%) | 35 (14.8%) | 0.87 (0.55-1.36) | .534 |
| >23.5 | 431 (20.6%) | 292 (28.3%) | 1.15 (0.96-1.37) | .131 | 347  (19.5%) | 264 (28.5%) | 1.32 (1.07-1.62) | .008 |  | 650  (22.8%) | 73 (26.7%) | 1.13 (0.84-1.51) | .419 | 548 (22.2%) | 63 (26.7%) | 1.08 (0.78-1.50 | .628 |
| Current smoker | N = 2105 | N = 1037 |  |  | N = 1781 | N = 926 |  |  |  | N = 2868 | N = 274 |  |  | N = 2471 | N = 236 |  |  |
| No | 1881 (89.4%) | 889 (85.7%) | Reference |  | 1599 (89.8%) | 802 (86.6%) | Reference |  |  | 2549 (88.9%) | 221 (80.7%) | Reference |  | 2211 (89.5%) | 190 (80.5%) | Reference |  |
| Yes | 224 (10.6%) | 148 (14.3%) | 1.4 (1.12-1.75) | .003 | 182  (10.2%) | 124 (13.4%) | 1.03 (0.79-1.36) | .817 |  | 319  (11.1%) | 53 (19.3%) | 1.92 (1.39-2.64) | <.001 | 260 (10.5%) | 46 (19.5%) | 1.75 (1.18-2.59) | .005 |
| COVID-19 vaccination | N = 2105 | N = 1037 |  |  | N = 1781 | N = 926 |  |  |  | N = 2868 | N = 274 |  |  | N = 2471 | N = 236 |  |  |
| Unvaccinated | 134  (6.4%) | 89  (8.6%) | Reference |  | 104  (5.8%) | 73  (7.9%) | Reference |  |  | 200  (7.0%) | 23  (8.4%) | Reference |  | 159  (6.4%) | 18  (7.6%) | Reference |  |
| One dose | 81  (3.8%) | 34  (3.3%) | 0.63 (0.39-1.02) | .062 | 66  (3.7%) | 29  (3.1%) | 0.72 (0.41-1.29) | .273 |  | 101  (3.5%) | 14  (5.1%) | 1.21 (0.59-2.44) | .604 | 82  (3.3%) | 13  (5.5%) | 1.47 (0.68-3.17) | .329 |
| Two or more dose | 1890 (89.8%) | 914 (88.1%) | 0.73 (0.55-0.96) | .026 | 1611 (90.5%) | 824  (89.0%) | 0.69 (0.49-0.97) | .035 |  | 2567 (89.5%) | 237 (86.5%) | 0.8 (0.51-1.26) | .341 | 2230 (90.2%) | 205 (86.9%) | 0.80 (0.48-1.34) | .396 |
| Diseases | N = 2105 | N = 1037 |  |  |  |  |  |  |  | N = 2868 | N = 274 |  |  |  |  |  |  |
| AR+AS | 828 (39.3%) | 402 (38.8%) | Reference |  |  |  |  |  |  | 1110  (38.7%) | 120 (43.8%) | Reference |  |  |  |  |  |
| AR | 1277 (60.7%) | 635 (61.2%) | 1.02 (0.88-1.19) | .758 |  |  |  |  |  | 1758 (61.3%) | 154 (56.2%) | 0.81 (0.63-1.04) | .099 |  |  |  |  |
| Total VAS^f^ score of AR symptoms | N = 1868 | N = 966 |  |  | N = 1781 | N = 926 |  |  |  | N = 2585 | N = 249 |  |  | N = 2471 | N = 236 |  |  |
| <15 | 1658 (88.8%) | 824 (85.3%) | Reference |  | 1581 (88.8%) | 789 (85.2%) | Reference |  |  | 2280 (88.2%) | 202 (81.1%) | Reference |  | 2178 (88.1%) | 192 (81.4%) | Reference |  |
| >=15 | 210 (11.2%) | 142 (14.7%) | 1.36 (1.08-1.71) | .008 | 200  (11.2%) | 137 (14.8%) | 1.07 (0.83-1.37) | .618 |  | 305 (11.8%) | 47 (18.9%) | 1.74 (1.24-2.44) | .001 | 293 (11.9%) | 44 (18.6%) | 1.52 (1.06-2.17) | .021 |
| AIT | N = 1984 | N = 994 |  |  | N = 1781 | N = 926 |  |  |  | N = 2718 | N = 260 |  |  | N = 2471 | N = 236 |  |  |
| No | 1010 (50.9%) | 753 (75.8%) | Reference |  | 902  (50.6%) | 701 (75.7%) | Reference |  |  | 1592 (58.6%) | 171 (65.8%) | Reference |  | 1446 (58.5%) | 157 (66.5%) | Reference |  |
| Yes | 974 (49.1%) | 241 (24.2%) | 0.33 (0.28-0.39) | <.001 | 879  (49.4%) | 225 (24.3%) | 0.67 (0.55-0.82) | <.001 |  | 1126 (41.4%) | 89 (34.2%) | 0.74 (0.56-0.96) | .024 | 1025 (41.5%) | 79 (33.5%) | 0.93 (0.68-1.29) | .670 |
| OMA | N = 2105 | N = 1037 |  |  |  |  |  |  |  | N = 2868 | N = 274 |  |  |  |  |  |  |
| No | 1807 (85.8%) | 913  (88.0%) | Reference |  |  |  |  |  |  | 2480 (86.5%) | 240 (87.6%) | Reference |  |  |  |  |  |
| Yes | 298 (14.2%) | 124  (12.0%) | 0.82 (0.66-1.03) | .089 |  |  |  |  |  | 388  (13.5%) | 34 (12.4%) | 0.91 (0.62-1.32) | .604 |  |  |  |  |

^a^RT: recovery time.

^b^HO: hospitalization.

^c^ER: emergency department visit.

^d^BMI: body mass index.

^e^VAS: visual analog scale.

| Table S6. Comparison of demographic, clinical, and COVID-19 characteristics between asthmatic patients in OMA+RMT and RMT groups. | | | |
| --- | --- | --- | --- |
|  | OMA+RMT  (N = 229) | RMT  (N = 589) | P-value |
| **Gender(male, %)^a^** | 89 (38.86) | 221 (37.52) | .722 |
| **Body mass index^b^** | 22.65 (20.20,24.61) | 23.23 (20.56,26.11) | .054 |
| **Age(years)^a^** |  |  |  |
| <18 (%) | 21 (9.17) | 62 (10.53) | .564 |
| ≥18 (%) | 208 (90.82) | 527 (89.47) |  |
| **Disease^a^** |  |  |  |
| AR | 0 (0.00) | 0 (0.00) | .055 |
| AS | 66 (28.82) | 132 (22.41) |  |
| AR and AS | 163 (71.18) | 457 (77.59) |  |
| **COVID-19 vaccination^a^** |  |  |  |
| Not vaccinated | 37 (16.16) | 58 (9.85) | .132 |
| One dose | 10 (4.37) | 21 (3.56) |  |
| Two doses | 58 (25.33) | 156 (26.49) |  |
| Three doses | 120 (52.40) | 344 (58.40) |  |
| Four doses | 4 (1.75) | 10 (1.70) |  |
| **The dosage of daily inhaled corticosteroid^a^** |  |  |  |
| Low dosage | 39 (17.03) | 233 (39.56) | <.001^c^ |
| Moderate dosage | 85 (37.11) | 240 (40.75) |  |
| High dosage | 105 (45.85) | 116 (19.69) |  |
| **Pre-infection asthma control condition^a^** |  |  |  |
| Well controlled | 153 (67.11) | 379 (64.35) | .195 |
| Not well controlled | 61 (26.75) | 150 (25.47) |  |
| Poor controlled | 14 (6.14) | 60 (10.18) |  |
| **Number of COVID-19 symptoms^a^** |  |  |  |
| None | 14 (6.11) | 12 (2.04) | .008^c^ |
| One | 21 (9.17) | 46 (7.81) |  |
| Two | 28 (12.22) | 49 (8.32) |  |
| Three | 27 (11.79) | 70 (11.88) |  |
| Four or more | 139 (60.70) | 412 (69.95) |  |
| **Symptoms of COVID-19 infection^a^** |  |  |  |
| Sore throat | 60 (26.20) | 237 (40.24) | <.001 |
| Dry cough | 53 (23.14) | 183 (31.07) | .025 |
| Productive cough | 113 (49.34) | 304 (51.61) | .560 |
| Chill | 69 (30.13) | 190 (32.26) | .557 |
| Fever | 180 (78.60) | 446 (75.72) | .383 |
| Muscle ache | 108 (78.60) | 321 (54.50) | .059 |
| Dizziness | 40 (17.46) | 134 (22.75) | .097 |
| Headache | 85 (37.11) | 211 (35.82) | .729 |
| Diarrhea | 15 (6.55) | 85 (14.43) | .002 |
| Stuffy nose | 61 (26.63) | 212 (35.99) | .011 |
| Runny nose | 50 (21.83) | 178 (30.22) | .016 |
| Chest tightness | 40 (17.46) | 140 (23.77) | .051 |
| Fatigue | 98 (42.79) | 250 (42.44) | .928 |
| Reduced or lost sense of taste/smell | 57 (24.89) | 157 (26.65) | .606 |
| Difficulty breathing | 19 (8.30) | 95 (16.13) | .004 |
| Chest pain | 10 (4.37) | 39 (6.62) | .222 |
| Rash | 9 (3.93) | 23 (3.90) | .987 |
| Heart palpitations | 19 (8.30) | 68 (11.55) | .176 |
| Joint pain | 52 (22.70) | 166 (28.18) | .112 |
| **COVID-19 infection requiring emergency visit or hospitalization^a^** | 21 (9.17) | 75 (12.73) | .155 |
| **Time required to recover to the pre-infection state^a^** | N = 229 | N = 554 |  |
| Within a week | 40 (17.46) | 95 (17.15) | .026^c^ |
| 1-2 weeks | 60 (26.20) | 99 (17.87) |  |
| 2-3weeks | 42 (18.34) | 92 (16.61) |  |
| More than 3 weeks | 87 (37.99) | 265 (47.83) |  |

^a^data that are expressed as percentage (%).

^b^data that are expressed as median (interquartile range).

^c^The p-values of chi-square analyses were validated by using Bonferroni correction.

| **Table S7. Comparison of demographic, clinical, and COVID-19 characteristics between patients in AIT+RMT and AIT+OMA+RMT groups.** | | | | | | | | | |
| --- | --- | --- | --- | --- | --- | --- | --- | --- | --- |
|  | **Total** | | | **Children** | | | **Adult** | | |
|  | **AIT+RMT group**  **(N = 1057)** | **AIT+OMA +RMT group**  **（N = 394)** | **P-value** | **AIT+RMT group**  **(N = 648)** | **AIT+OMA+RMT**  **group**  **(N = 117)** | **P-value** | **AIT+RMT group**  **(N = 409)** | **AIT+OMA+RMT**  **group**  **(N = 277)** | **P-value** |
| **Gender(male, %)^a^** | 613 (57.99) | 257 (65.23) | .012 | 450 (69.44) | 84 (71.79) | .610 | 163 (39.85) | 173 (62.45) | <.001 |
| **Body mass index^b^** | 20.20 (16.30,23.72) | 21.04 (18.74,23.36) | <.001 | 17.18 (15.15,21.10) | 17.14 (14.95,21.55) | .945 | 23.05 (20.78,25.52) | 23.24  (21.11,25.00) | .847 |
| **Age(years)^a^** |  |  |  |  |  |  |  |  |  |
| <18 (%) | 648 (61.31) | 117 (29.70) | <.001 | 648 (100.00) | 117 (100.00) | - | 0 (0.00) | 0 (0.00) | - |
| ≥18 (%) | 409 (38.69) | 277 (70.30) |  | 0.00 (0.00) | 0 (0.00) |  | 409 (100.00) | 277 (100.00) |  |
| **Smoking history^a^** |  |  |  |  |  |  |  |  |  |
| Current smoker | 21 (1.99) | 7 (1.78) | .796 | 0 (0.00) | 0 (0.00) | - | 21 (5.13) | 7 (2.53) | .090 |
| **Disease^a^** |  |  |  |  |  |  |  |  |  |
| AR | 654 (61.87) | 99 (25.13) | <.001 | 396 (61.11) | 50 (42.74) | <.001 | 258 (63.08) | 49 (17.69) | <.001 |
| AS | 74 (7.01) | 162 (41.12) |  | 51 (7.87) | 11 (9.40) |  | 23 (5.62) | 151 (54.51) |  |
| AR and AS | 329 (31.13) | 133 (33.76) |  | 201 (31.02) | 56 (47.86) |  | 128 (31.30) | 77 (27.80) |  |
| **Pre-infection asthma control condition^a^** | N = 400 | N = 295 |  | N = 251 | N = 67 |  | N = 149 | N = 228 |  |
| Well controlled | 342 (85.50) | 252 (85.42) | .801 | 230 (91.63) | 61 (91.04) | .881 | 112 (75.17) | 191 (83.77) | .081 |
| Not well controlled | 44 (11.00) | 35 (11.86) |  | 16 (6.37) | 4 (5.97) |  | 28 (18.79) | 31 (13.60) |  |
| Poor controlled | 14 (3.50) | 8 (2.71) |  | 5 (1.99) | 2 (2.99) |  | 9 (6.04) | 6 (14.81) |  |
| **Allergic rhinitis symptoms score^a^** | N = 906 | N = 201 |  | N = 566 | N = 93 |  | N = 339 | N = 108 |  |
| Stuffy nose (VAS^d^ ≥ 5) | 145 (16.00) | 31 (15.42) | .823 | 28 (4.94) | 4 (4.30) | .654 | 66 (19.47) | 21 (19.44) | .996 |
| Sneezing (VAS ≥ 5) | 104 (11.48) | 23 (11.44) | .988 | 21 (3.71) | 4 (4.30) | .921 | 52 (15.34) | 17 (15.74) | .920 |
| Runny nose (VAS ≥ 5) | 87 (9.60) | 23 (11.44) | .430 | 17 (3.00) | 3 (3.23) | .970 | 41 (12.09) | 16 (14.81) | .460 |
| **COVID-19 Vaccination^a^** |  |  |  |  |  |  |  |  |  |
| Not vaccinated | 67 (6.34) | 25 (6.34) | <.001 | 45 (6.94) | 9 (7.69) | .219 | 22 (5.38) | 16 (5.78) | <.001 |
| One dose | 41 (3.87) | 43 (10.75) |  | 26 (4.01) | 5 (4.27) |  | 15 (3.67) | 38 (13.72) |  |
| Two doses | 622 (58.85) | 196 (49.75) |  | 533 (82.25) | 95 (81.20) |  | 89 (21.76) | 102 (36.82) |  |
| Three doses | 317 (30.00) | 121 (30.71) |  | 44 (6.79) | 7 (5.98) |  | 273 (66.75) | 116 (41.88) |  |
| Four doses | 10 (0.95) | 6 (1.52) |  | 0 (0.00) | 1 (0.85) |  | 10 (2.44) | 5 (1.81) |  |
| **The number of symptoms of COVID-19 infection^a^** |  |  |  |  |  |  |  |  |  |
| None | 65 (6.15) | 47 (11.93) | <.001 | 53 (8.17) | 8 (6.84) | .473 | 12 (2.93) | 39 (14.08) | <.001 |
| One | 205 (19.39) | 54 (13.71) |  | 172 (26.54) | 27 (23.08) |  | 33 (8.07) | 27 (9.74) |  |
| Two | 176 (16.65) | 107 (27.16) |  | 134 (20.68) | 21 (17.95) |  | 42 (10.27) | 86 (31.05) |  |
| Three | 135 (12.77) | 70 (17.77) |  | 96 (14.81) | 16 (13.68) |  | 39 (9.53) | 54 (19.49) |  |
| Four or more | 476 (45.03) | 116 (29.44) |  | 193 (29.78) | 45 (38.46) |  | 283 (69.19) | 71 (25.63) |  |
| **Symptoms of COVID-19 infection^a^** |  |  |  |  |  |  |  |  |  |
| Sore throat | 328 (31.03) | 121 (30.71) | .906 | 141 (21.76) | 36 (30.77) | .033 | 187 (45.72) | 85 (30.69) | <.001 |
| Cough | 248 (23.46) | 90 (22.84) | .804 | 115 (17.75) | 24 (20.51) | .475 | 133 (32.52) | 66 (23.83) | .014 |
| Productive cough | 361 (34.15) | 165 41.88) | .006 | 151 (23.30) | 31 (26.50) | .455 | 210 (51.34) | 134 (48.38) | .445 |
| Chill | 195 (18.45) | 46 (11.67) | .002 | 59 (9.10) | 14 (11.97) | .332 | 136 (33.25) | 32 (11.55) | <.001 |
| Fever | 851 (80.51) | 201 (51.02) | <.001 | 530 (81.79) | 98 (83.76) | .609 | 321 (78.48) | 103 (37.18) | <.001 |
| Muscle ache | 323 (30.59) | 152 (38.58) | .004 | 109 (16.82) | 37 (31.62) | <.001 | 214 (52.32) | 115 (41.52) | .005 |
| Dizziness | 234 (22.14) | 47 (11.93) | <.001 | 136 (20.99) | 23 (19.66) | .744 | 98 (23.96) | 24 (8.66) | <.001 |
| Headache | 319 (30.18) | 75 (19.03) | <.001 | 161 (24.85) | 35 (29.91) | .248 | 158 (38.63) | 40 (14.44) | <.001 |
| Diarrhea | 77 (7.28) | 18 (4.57) | .063 | 22 (3.40) | 4 (3.42) | .990 | 55 (13.44) | 14 (5.05) | <.001 |
| Stuffy nose | 276 (26.11) | 67 (17.01) | <.001 | 126 (19.44) | 26 (22.22) | .488 | 150 (36.67) | 41 (14.80) | <.001 |
| Runny nose | 230 (21.75) | 43 (10.91) | <.001 | 110 (16.97) | 20 (17.09) | .975 | 120 (29.34) | 23 (8.30) | <.001 |
| Chest tightness | 80 (7.57) | 34 (8.62) | .504 | 13 (2.01) | 2 (1.71) | .831 | 67 (16.38) | 27 (9.75) | .013 |
| Fatigue | 284 (26.87) | 72 (18.27) | .001 | 102 (15.74) | 25 (21.36) | .132 | 182 (44.50) | 47 (16.96) | <.001 |
| Decreased sense of taste/smell | 183 (17.31) | 38 (9,64) | <.001 | 46 (7.10) | 9 (7.69) | .819 | 137 (33.50) | 29 (10.47) | <.001 |
| Difficulty breathing | 52 (4.92) | 16 (4.06) | .491 | 7 (1.08) | 4 (3.41) | .051 | 45 (11.00) | 12 (4.33) | .002 |
| Chest pain | 37 (3.50) | 9 (2.28) | .240 | 6 (0.93) | 3 (2.56) | .130 | 31 (7.58) | 7 (2.53) | .005 |
| Rash | 29 (2.74) | 14 (3.55) | .419 | 15 (2.31) | 4 (3.41) | .480 | 14 (3.42) | 10 (3.61) | .896 |
| Heart palpitations | 43 (4.07) | 15 (3.80) | .821 | 3 (0.46) | 2 (1.71) | .124 | 41 (10.02) | 13 (4.69) | .011 |
| Joint pain | 161 (15.23) | 88 (22.33) | .001 | 48 (7.41) | 17 (14.53) | .011 | 113 (27.62) | 71 (25.63) | .562 |
| **COVID-19 infection requiring emergency visit or hospitalization^a^** | 77 (7.28) | 18 (4.56) | .063 | 37 (5.71) | 8 (6.84) | .663 | 40 (9.78) | 10 (3.61) | .002 |
| **Time required to recover to the pre-infection state^a^** | N = 1026 | N = 394 |  | N=640 | N=117 |  | N=386 | N=277 |  |
| Within a week | 441 (42.98) | 183 (46.45) | .081 | 381 (59.53) | 74 (63.24) | .861 | 68 (17.62) | 113 (40.79) | <.001 |
| 1-2 weeks | 231 (22.51) | 99 (25.12) |  | 157 (24.53) | 25 (21.37) |  | 77 (19.95) | 78 (28.16) |  |
| 2-3weeks | 134 (13.06) | 53 (13.45) |  | 55 (8.59) | 9 (7.69) |  | 74 (19.17) | 36 (13.00) |  |
| More than 3 weeks | 214 (20.86) | 59 (14.97) |  | 47 (7.34) | 9 (7.69) |  | 167 (43.26) | 50 (18.05) |  |

^a^data that are expressed as percentage (%).

^b^data that are expressed as median (interquartile range).

^c^The p-values of chi-square analyses were validated by using Bonferroni correction.

^d^VAS: visual analog scale.

| Table S8. Univariate and multivariate logistic analysis to identify the factors contributing to COVID-19 outcomes for subjects with AIT | | | | | | | | | | | | | | | | | |
| --- | --- | --- | --- | --- | --- | --- | --- | --- | --- | --- | --- | --- | --- | --- | --- | --- | --- |
|  | To identify the factors contributing to RT^a^ < 3w (outcome 1) | | | | | | | |  | To identify the factors contributing to Non-HO^b^ or ER^c^ (outcome 2) | | | | | | | |
|  | Univariate logistic analysis | | | | Multivariate logistic analysis | | | |  | Univariate logistic analysis | | | | Multivariate logistic analysis | | | |
| Variables | RT < 3w  (N, %) | RT ≥ 3w  (N, %) | OR  (95%CI) | P- value | RT < 3w  (N, %) | RT ≥ 3w  (N, %) | Adjusted-OR  (95%CI) | P- value |  | Non-HO or ER  (N, %) | HO or ER  (N, %) | OR  (95%CI) | P- value | Non-HO or ER  (N, %) | HO or ER  (N, %) | Adjusted-OR  (95%CI) | P- value |
| Age (years) | N = 1178 | N = 273 |  |  | N = 1174 | N = 272 |  |  |  | N = 1356 | N = 95 |  |  |  |  |  |  |
| <18 | 709 (60.2%) | 56 (20.5%) | Reference |  | 707 (60.2%) | 55 (20.2%) |  |  |  | 720  (53.1%) | 45 (47.4%) |  |  |  |  |  |  |
| >=18 | 469 (39.8%) | 217 (79.5%) | 5.86 (4.27-8.04) | <.001 | 467 (39.8%) | 217 (79.8%) | 5.56 (3.72-8.29) | <.001 |  | 636  (46.9%) | 50 (52.6%) | 1.26 (0.83-1.91) | .281 |  |  |  |  |
| Gender | N = 1177 | N = 273 |  |  | N = 1174 | N = 272 |  |  |  | N = 1355 | N = 95 |  |  |  |  |  |  |
| Female | 423 (35.9%) | 157 (57.5%) | Reference |  | 422 (35.9%) | 156 (57.4%) |  |  |  | 544  (40.1%) | 36 (37.9%) |  |  |  |  |  |  |
| Male | 754 (64.1%) | 116 (42.5%) | 0.41 (0.32-0.54) | <.001 | 752 (64.1%) | 116 (42.6%) | 1.22 (0.77-1.94) | .399 |  | 811  (59.9%) | 59 (62.1%) | 1.10 (0.72-1.69) | .665 |  |  |  |  |
| BMI^d^ | N = 1175 | N = 272 |  |  | N = 1174 | N = 272 |  |  |  | N = 1352 | N = 95 |  |  |  |  |  |  |
| 18-23.5 | 547 (46.6%) | 160 (58.8%) | Reference |  | 547 (46.6%) | 160 (58.8%) |  |  |  | 661  (48.9%) | 46 (48.4%) |  |  |  |  |  |  |
| <18 | 459 (39.1%) | 43 (15.8%) | 0.32 (0.22-0.46) | <.001 | 459 (39.1%) | 43 (15.8%) | 0.79 (0.51-1.23) | .300 |  | 471  (34.8%) | 31 (32.6%) | 0.95 (0.59-1.51) | .816 |  |  |  |  |
| >23.5 | 169 (14.4%) | 69 (25.4%) | 1.40 (1.00-1.94) | .048 | 168 (14.3%) | 69 (25.4%) | 1.29 (0.90-1.86) | .164 |  | 220  (16.3%) | 18 (18.9%) | 1.18 (0.67-2.07) | .575 |  |  |  |  |
| Current smoker | N = 1178 | N = 273 |  |  | N = 1174 | N = 272 |  |  |  | N = 1356 | N = 95 |  |  |  |  |  |  |
| No | 1087 (92.3%) | 238 (87.2%) | Reference |  | 1083 (92.2%) | 237 (87.1%) |  |  |  | 1239 (91.4%) | 86 (90.5%) |  |  |  |  |  |  |
| Yes | 91 (7.7%) | 35 (12.8%) | 1.76 (1.16-2.66) | .008 | 91 (7.8%) | 35 (12.9%) | 1.22 (0.77-1.94) | .399 |  | 117  (8.6%) | 9  (9.5%) | 1.11 (0.54-2.26) | .777 |  |  |  |  |
| COVID-19 Vaccination | N = 1178 | N = 273 |  |  | N = 1174 | N = 272 |  |  |  | N = 1356 | N = 95 |  |  |  |  |  |  |
| Unvaccinated | 73 (6.2%) | 19 (7.0%) | Reference |  | 71  (6.0%) | 19  (7%) |  |  |  | 86  (6.3%) | 6  (6.3%) |  |  |  |  |  |  |
| One dose | 71 (6.0%) | 13 (4.8%) | 0.70 (0.32-1.53) | .375 | 71  (6.0%) | 13  (4.8%) | 0.62 (0.27-1.42) | .255 |  | 76  (5.6%) | 8  (8.4%) | 1.51 (0.50-4.54 | .465 |  |  |  |  |
| Two or more dose | 1034 (87.8%) | 241 (88.3%) | 0.90 (0.53-1.51) | .680 | 1032 (87.9%) | 240 (88.2%) | 0.77 (0.43-1.36) | .362 |  | 1194 (88.1%) | 81 (85.3%) | 0.97 (0.41-2.29) | .949 |  |  |  |  |
| Diseases | N = 1178 | N = 273 |  |  |  |  |  |  |  | N = 1356 | N = 95 |  |  | N = 1356 | N = 95 |  |  |
| AR+AS | 369 (31.3%) | 93 (34.1%) | Reference |  |  |  |  |  |  | 422  (31.1%) | 40 (42.1%) |  |  | 422  (31.1%) | 40 (42.1%) |  |  |
| AS | 204 (17.3%) | 32 (11.7%) | 0.62 (0.40-0.96) | .033 |  |  |  |  |  | 230  (17.0%) | 6  (6.3%) | 0.28 (0.11-0.66) | .004 | 230  (17.0%) | 6  (6.3%) | 0.70 (0.45-1.09) | .011 |
| AR | 605 (51.4%) | 148 (54.2%) | 0.97 (0.73-1.30) | .840 |  |  |  |  |  | 704  (51.9%) | 49 (51.6%) | 0.73 (0.48-1.13) | .164 | 704  (51.9%) | 49 (51.6%) | 0.31  (0.13-0.76) | .116 |
| AIT | N = 1178 | N = 273 |  |  | N = 1174 | N = 272 |  |  |  | N = 1356 | N = 95 |  |  | N = 1356 | N = 95 |  |  |
| AIT | 843 (71.6%) | 214 (78.4%) | Reference |  | 841 (71.6%) | 213 (78.3%) |  |  |  | 980  (72.3%) | 77 (81.1%) |  |  | 980  (72.3%) | 77 (81.1%) |  |  |
| AIT+OMA | 335 (28.4%) | 59 (21.6%) | 0.69 (0.51-0.95) | .023 | 333 (28.4%) | 59 (21.7%) | 0.44 (0.31-0.62) | <.001 |  | 376  (27.7%) | 18 (18.9%) | 0.61 (0.36-1.03) | .065 | 376  (27.7%) | 18 (18.9%) | 0.73 (0.42-1.28) | .277 |

^a^RT: recovery time.

^b^HO: hospitalization.

^c^ER: emergency department visit.

^d^BMI: body mass index.
