# Supplementary material for: Protective Effect of Allergen Immunotherapy in Patients With Allergic Rhinitis and Asthma Against COVID-19 Infection: Observational, Nationwide, and Multicenter Study
Source: JMIR Public Health Surveill. 2024 Oct 16;10:e50846. doi: 10.2196/50846 (PMC11498206; doi:10.2196/50846)
Supplement: Multimedia Appendix 2 [file publichealth-v10-e50846-s002.docx]

The detail name list of contributors to our work

We sincerely thank all the health care providers fighting against this public crisis and all the participants involved in the study. We thank the hospital staff for their efforts in collecting the information. We are indebted to the coordination of Drs Juntao Feng (Xiangya Hospital Central South University), Wei Song (Dalian Women and Children's Medical Group), Xiang Gao (The Affiliated Hospital of Qingdao University), Liang Chen (Xiamen Chang Gung Hospital), Linfeng Wang (The First Affiliated Hospital of Ningbo University), Lihua Lin (The First Affiliated Hospital of Xiamen University), Fucheng An (Beijing Mentougou District Hospital), Yan Qin, Tingting Ma (Beijing Shijitan Hospital, Capital Medical University), Yi Zhang, Yanmin Bao (Shenzhen Children's Hospita), Kang Xu, Yanlian Zhao (Shenzhen Luohu Hospital Group Luohu People's Hospital. The third Affiliated Hospital of Shenzhen University), Huan Zhang, Hao Chen (Tongji Hospital, Tongji Medical College, Huazhong University of Science & Technology), Weiquan Liang, Huijuan Zhang (Civil Aviation General Hospital), Jingmin Deng (The First Affiliated Hospital of Guangxi Medical University), Yuemei Sun (Yantai Yuhuangding Hospital), Liping Guan, Diannan Miao (Xinqiao Hospital，Army Medical University), Yungang Yang (The First Affiliated Hospital of Xiamen University), Yan Zhao (The First Affiliated Hospital of Harbin Medical University), Wei Tang (Ruijin Hospital, Shanghai Jiaotong University School of Medicine), Yi Liang, Xiuyan Qu (Zhongshan City People's Hospital), Yao Tang (The First Affiliated Hospital of Harbin Medical University), Lihui Wang (The First Bethune Hospital of Jilin University), Rongfei Zhu (Tongji Hospital, Tongji Medical College, Huazhong University of Science & Technolog),Fang Chen (Zhejiang Provincial Hospital of Chinese Medicine), Min Xie (Tongji Hospital, Tongji Medical College, Huazhong University of Science & Technology), Xiaojun Qian (The Third People's Hospital of Hefei), Weiping Tan (The First Affiliated Hospital, Sun Yat-sen University), Ruonan Chai, Hangming Dong (Nanfang Hospital), Yuebin Wang (The Third People's Hospital of Chengdu), Dan Zhang (The Fourth Affiliated Hospital Zhejiang University School of Medicine), Yangli Liu (The First Affiliated Hospital, Sun Yat-sen University), Nan Huang (Tongji Hospital, Tongji Medical College, Huazhong University of Science & Technology), Zhanying Ma (Dongguan Maternal And Child Health Care Hospital), Xuefang Zheng (Dongguan Maternal And Child Health Care Hospital), Jianhua Zhu (The First Affiliated Hospital, Sun Yat-sen University), Lixia Huang (The First Affiliated Hospital, Sun Yat-sen University), Shaoli Li (The First Affiliated Hospital, Sun Yat-sen University), Gengpeng Lin (The First Affiliated Hospital, Sun Yat-sen University), Xitao Zhang (Shantou Hospital of TCM), Wo Yao (The Second Affiliated Hospital Zhejiang University School of Medicine), Lingling Chen (Taizhou Hospital of Zhejiang Province), Bing Lu, Xueyan Wang (Beijing Shijitan Hospital, Capital Medical University), Hongmei Yao (Guizhou Provincial People'S Hospital), Jinling Liu (Children's Hospital of Zhejiang University School of Medicine), Wei Wang (Shenyang Children's Hospital), Yingshuo Wang (Children's Hospital of Zhejiang University School of Medicine), Qingyu Wei (Shengjing Hospital of China Medical University), Ying Ga (Dalian Women and Children's Medical Group), Lin He (Chongqing University Fuling Hospital), Jing Liu (The Fifth Affiliated Hospital Sun Yat-sen University), Yingbin Mai (Shantou Longhu Hospital), Bin Li (Huangshi Central Hospital), Fengxia Liu (Weifang People's Hospital), Huijuan Xu (The Second Affiliated Hospital of Shantou University Medical College), Caiye Jiang (Xiamen Chang Gung Hospital), Shuang Suo (Beijing Mentougou District Hospital), Xueyan Wang (The Second Hospital of Tianjin Medical University), Boyun Yang (The Second Affiliated Hospital Zhejiang University School of Medicine), Jinghua Yang (Guangdong Provincial Hospital of Traditional Chinese Medicine), Xiao Yong (Weihai Municipal Hospital), Yiwen You (Affiliated Hospital of Nantong University), Zenghong He, Bohui Li (The Second Affiliated Hospital Zhejiang University School of Medicine), Zhiwei Lu (Shenzhen Children's Hospital), Ying Song (Heilongjiang Provincial Hospital), Xiufang Xiong, Mingzhou Zhang (Xinqiao Hospital，Army Medical University), Jianxue Bai, Ran Diao (The Second Affiliated Hospital Zhejiang University School of Medicine), Li Liu (Civil Aviation General Hospital), Ling Wang (Hainan General Hospital (Hainan Affiliated Hospital of Hainan Medical University), Zhumei Chen (The Fifth Affiliated Hospital Sun Yat-sen University), Junan Liu (Xiamen Chang Gung Hospital), Bing Zhang (The Second Affiliated Hospital Zhejiang University School of Medicine), YaDong Gao (Zhongnan Hospital of Wuhan University), Lingfei Kong (The First Hospital of China Medical University), Xingli Lei, Mingfeng LI, Guanghui Liu (Zhongnan Hospital of Wuhan University), Jianbin Lu (Zhangjiagang First People's Hospital), Jian Luo (Xiamen Chang Gung Hospital), Yuanhang Nie, Ningbo Tang (Yantai Yuhuangding Hospital), Feng Yang (Xiamen Chang Gung Hospital), Yaqi Yang (Tongji Hospital, Tongji Medical College, Huazhong University of Science & Technology), Qingqing Yu (The First People'S Hospital of Foshan), Hanrong Zhong (The First People'S Hospital of Foshan), Yunlian Zhou (Children's Hospital of Zhejiang University School of Medicine), Junfeng Zhou (Wenling Maternal and Child Health Care Hospital), Jianhua Cao (Zhaluteqi People's Hospital), Jing Chen (Affiliated Hospital of Nantong University), Zhimin Chen (Children's Hospital of Zhejiang University School of Medicine), Hong Dai, Qiangwei Dai (Shenzhen Children's Hospital), Feifei Dou (Yantai Yuhuangding Hospital), Ping He, Yong He (The Affiliated Hospital of Medical School,Ningbo University), Huaqiong Huang (The Second Affiliated Hospital Zhejiang University School of Medicine), Qianqian Lei (The Fifth Affiliated Hospital Sun Yat-sen University), Quansheng Li (Shengjing Hospital of China Medical University), Shuxian Li (Children's Hospital of Zhejiang University School of Medicine), Xingwang Li (Civil Aviation General Hospital), Xiaoping Li, Nana Sun (Nanfang Hospital), Jianxin Xiao (The First People'S Hospital of Foshan), Jihong Yang (Shenzhen Luohu Hospital Group Luohu People's Hospital. The third Affiliated Hospital of Shenzhen University), Ruili Yu (Beijing Shijitan Hospital, Capital Medical University), Wenchao Zhang (Henan Provincial People's Hospital), Yu Zhang (ZiBo Central Hospital), Huafei Zhou (LuQiao School District,Taizhou Hospital), Ning Zhu (The Second Hospital of Dalian Medical University), Xiaoming Zhu (Shengjing Hospital of China Medical University), Chun Chang (Peking University Third Hospital), Mo Xian (The First Affiliated Hospital of Guangzhou Medical University), Jiaxing Xie (The First Affiliated Hospital of Guangzhou Medical University), Wanjun Wang (The First Affiliated Hospital of Guangzhou Medical University), Xu Shi (The First Affiliated Hospital of Guangzhou Medical University), Shunkai Huang (The First Affiliated Hospital of Guangzhou Medical University), Shiyue Li (The First Affiliated Hospital of Guangzhou Medical University), which greatly facilitated the collection of patients’ data. We thank Zhaowei Yang, Jia Nan, Wanyi Fu, Mo Xian, Junfeng Huang, Zikai Lin and Mei Jiang (Guangzhou Institute of Respiratory Health) for the suggestion on the manuscript drawing and statistical analysis. We thank Yubiao Guo, Xiaonan Song, Fangmei Lin, Siyang Yao,and Li He (Guangzhou Institute of Respiratory Health) for their dedication to data entry and verification.
